# Hope as experienced by people with acquired brain injury in a rehabilitation—or recovery process: a qualitative systematic review and thematic synthesis

**DOI:** 10.3389/fresc.2024.1376895

**Published:** 2024-05-14

**Authors:** Camilla Højgaard Nejst, Chalotte Glintborg

**Affiliations:** ^1^ Neurorehabilitation Copenhagen, Municipality of Copenhagen, Copenhagen, Denmark; ^2^ Department of Communication and Psychology, Aalborg University, Aalborg, Denmark

**Keywords:** hope, acquired brain injury, rehabilitation, recovery, literature review, qualitative studies, thematic synthesis

## Abstract

**Background:**

There has been an increasing interest in the concept of hope within the field of brain injury rehabilitation. Existing reviews have nevertheless focused on stroke, leaving out the broad population of people with acquired brain injury (ABI). Furthermore a majority of the included studies in those reviews excluded the subgroup of people with communication difficulties, thus primarily giving voice to a select group of people with ABI.

**Methods:**

A qualitative systematic review was conducted with the purpose of systematically reviewing and thematically synthesise findings about hope as experienced by adults with ABI in a rehabilitation or recovery process. The search strategy included peer-reviewed qualitative studies published after 2000 in English or Scandinavian languages. Searches of EBSCO databases incorporating CINAHL, MEDLINE, and PsycINFO were conducted together with SocINDEX, Social Work Abstracts, Eric and Web of Science. Ten qualitative studies were included, and the Critical Appraisal Skills Program (CASP) was used for assessing the quality and relevance of the ten studies. Qualitative findings were synthesized using Thomas and Harden's methodology.

**Results:**

Through a thematic synthesis eleven subthemes were identified relating to experiences of hope. These were grouped into four analytical themes: (1) hope a two folded phenomenon; (2) time and temporality; (3) progress, goals and visibility and (4) the alliance; a balancing act requiring good communication skills.

**Conclusion:**

This review has shown that even though hope has both a positive and negative side to it, it is necessary as a driving force for people with ABI in terms of supporting them to keep going and not give up. Rehabilitation professionals are advised to embrace the ambiguity of hope, customizing the support of hope to each person with ABI. Attention is needed on how to make progress visible for persons with ABI during their rehabilitation process just as rehabilitation professionals should acknowledge the alliance with the person with ABI as a core component of rehabilitation. This requires a focus on professionals' communication skills if hope promoting relationships between professionals and persons with ABI are to be achieved.

## 1 Introduction

When thinking of hope, one might come across stories of the nurse Florence Nightingale, also called “the Lady with the Lamp”. Florence Nightingale is particularly known for walking through the infirmaries at night with a lamp in her hand—tending to the wounded, often with a comforting word on her lips. It is therefore often referenced that Florence Nightingale walked the dark corridors and spread hope and light to the patients (1).

Since the 1950s, the concept of hope has gained relevance across disciplines and research cultures such as positive psychology, psychiatry, and nursing research (2–4). Many researchers have depicted the meaning of hope to human beings and described hope in numerous ways (5–8). However, researchers across disciplines agree that hope is both universal and specific (concrete) (5, 8, 9). Universal hope, according to Hammer, can be described as “a general belief in the future and a safeguard for human being by illuminating life itself”, whereas specific hope is described by Hammer as “connected to time and object” (8). Snyder et al. (10) have worked on a definition of hope (within rehabilitation) where hope is described as a goal-directed cognitive motivational process focused on the importance of particularized hope and goals (10). In contrast, research on hope within the field of chronic illness is defined and described differently. The researcher Barnard for instance says: “hoping is a posture, not a motive for achievement of a particular goal. It is a mode of experiencing oneself in relation to reality and time” (11).

Earlier studies often focused on hopelessness rather than hope, especially for persons with mental illness. But as researchers began to expand their horizons, they ended up exploring the construct of hope across diverse illness contexts, including the chronically ill, critically ill and terminally ill (9). Later, also research within vulnerable groups such as people with dual diagnosis have been explored as to how hope is experienced by people with co-occurring mental health and substance use problems (12–14), just as hope within the population of patients with spinal cord injury has been explored in recent years (15–19). Hope is therefore a well-known theme in relation to serious illness and disability. Many anthropological and sociological studies have emphasized the despair that can follow chronic and serious conditions (20–25), often connected with lack of hope or the struggle for hope (26).

### 1.1 Hope and acquired brain injury

When a person acquires a brain injury, something happens not only to the brain and body, but also the anticipated future. Experiencing an ABI can thus be conceptualized as a “critical event” that disrupts the structure of everyday life, The sociologist Bury refers to this event as a form of biographical disruption (23). In other words, an unexpected interruption of an otherwise expected normal course of life.

An acquired brain injury may be caused by stroke, hemorrhages in the brain other than stroke, trauma, tumors (benign and malignant), infections, poisoning, lack of oxygen (e.g., by drowning accidents and cardiac arrest with successful resuscitation) etc. (27). The consequences of brain injury can be physical, cognitive, psychological, linguistic, and communicative and often have a major impact on a person's life and identity According to Cantor et al., persons with ABI live with two images of the self: “Who I am now” and “Who I was before” (28). This identity reconstruction process is a dynamic process of contraction and expansion in which the person with ABI strikes a tentative balance between new and old selves. This process might call for professional support to revise self-narratives and to validate the loss of some identities (e.g., working identity) (29).

The rehabilitation of people with acquired brain injury strives for a holistic approach, where the bio-psycho-social model forms the basis for all rehabilitation (30, 31). However, Danish research within brain injury rehabilitation has shown that rehabilitation continues to mostly focus on the rehabilitation of practical functions in everyday life (Activities of Daily Living), physical functions and vocational rehabilitation (29, 32, 33). Thus, rehabilitation is primarily aimed at physical and functional rehabilitation, and not a holistic focus where the person's existential and emotional situation is also considered. Within a bio-psycho-social rehabilitation framework, there has been an increasing interest in the concept of hope. Having a hopeful stance and being able to identify possible pathways into a meaningful future is essential in times of uncertainty and despair.

Nochi (34) found that hope of recovery was used as a strategy by persons who had sustained a traumatic brain injury to reduce this experience of “loss of self”. Kuipers and colleagues (35), have identified a need for clinically relevant research on hope, which can guide practitioners in fostering hope to enhance the experiences and outcomes for persons with ABI and their family members (35).

#### 1.1.1 Reviews on hope and stroke

In 2011, Bright and colleagues conducted a systematic review on hope after stroke (36). Nineteen articles were included, but only seven of these articles sought to explore hope (37–43). For the remaining 12 articles, hope was a key finding that was present when related topics such as recovery or quality of life were explored (36). Bright et al.'s review revealed that hope was complex and multidimensional. Moreover, hope was influenced by internal and external sources and had a positive impact on recovery following stroke. Their proposed conceptualization identified three attributes of hope: (1) an inner state, (2) outcome orientation, and (3) an active process (2, 36).

Soundy and colleagues published two reviews in 2014 (44, 45). The first being a narrative review focusing on factors promoting or hindering hope in persons with stroke or spinal cord injury. Moreover, it focused on identifying how (health) professionals could support the promotion of hope during rehabilitation (44). The second review proposed a framework for hope, arguing for a more generalized view of understanding why a certain hope exists or is identified by a patient (45).

Whereas Bright et al. included nineteen studies in their review, Soundy et al. ended up including ten studies. A total of four studies overlapped and were thus included in both reviews (37, 38, 43, 46). The above-mentioned reviews have contributed both in enhancing our understanding of hope. However, we still do not know much about the role of hope in persons following an ABI as both research teams only focused on people with stroke. Furthermore, more than half of the included studies in the three reviews either completely excluded persons with communication difficulties or only included persons if they didn't have pronounced communication difficulties. As a result, we know even less about experiences of hope when it comes to persons with communication difficulties (47).

Therefore, the purpose of this review is to capture the phenomenon of hope as experienced by the broad population of persons with ABI. Through a systematic search in relevant databases and a thematic synthesis, we aim to expand our understanding of hope as experienced and described by persons with acquired brain injury during a rehabilitation or recovery process. This knowledge can inform rehabilitation theory and practice further.

#### 1.1.2 Review question

The specific question guiding this review is: “How is hope (as a phenomenon) experienced in the rehabilitation or recovery process of persons with acquired brain injury”? The qualitative PICo worksheet adapted from Miller, S.A. (2001) was used as a framework for building the search strategy^1^ (48). The different search terms for each of the three blocks in PICo are presented in Table 1.

**Table 1:** PICo describing database search strategy.

Population	(stroke OR “brain injury” OR “brain injuries” OR “head injury” OR “head injuries” OR “head trauma” OR “traumatic brain injury” OR “traumatic brain injuries” OR TBI OR “brain trauma” OR “acquired brain injury” OR “acquired brain injuries” OR ABI OR “brain damage” OR “brain infarction” OR “cerebral infarction” OR apoplexia OR apoplexy OR “neurological impairment” OR “neurological impairments” OR “central nervous system neoplasm” OR “central nervous system neoplasms” OR “central nervous system tumor” OR “cerebral haemorrhage” OR “cerebrovascular accident” OR “encephalopathy” OR “subarachnoid haemorrhage”)
AND	
Phenomenon of interest	(hope OR hopeful OR hopefulness OR hopelessness OR despair OR “meaning-making” OR “meaning making” OR “meaning-making process” OR “meaning making process” OR optimism OR pessimism OR wish OR “wishful thinking”)
AND	
Context	(rehabilitation OR recovery OR “rehabilitation process” OR “recovery process” OR “rehabilitation journey” OR “recovery journey”)

## 2 Methods

A qualitative systematic literature review was conducted, and the approach and reporting were guided by the Enhancing Transparency in Reporting the Synthesis of Qualitative Research (ENTREQ) statement (49) and the PRISMA statement (50). To answer the research question, study results were synthesized, applying a thematic synthesis, as described by Thomas and Harden (51).

The research team conducting the search, screening and final inclusions of studies consist of the two authors (CHN and CG). The first author has several years of clinical experience within the field of ABI rehabilitation. The second author has done research within the field of ABI rehabilitation for more than 10 years and also holds clinical rehabilitation experience. Having a phenomenological standpoint, the authors are interested in exploring the lived experience of hope, thereby looking at hope from a first person perspective (persons with ABI).

### 2.1 Search strategy

A “subject-based strategy” was chosen since the approach has been shown to be more effective than the alternative (“research methodology-based strategy”) in retrieving qualitative patient-reported health-related quality of life research (52).

To develop relevant keywords (synonyms, near synonyms, broader and narrower concepts), pilot searches in selected databases were conducted (52, 53). The search was initially conducted in December 2021 with a follow up search in January 2023 (see Supplementary Appendices S1, S2 for search protocols) to ensure that any new studies meeting the inclusion criteria were included. However, no relevant studies were identified in this new search. The literature search included English and Scandinavian peer-reviewed articles published between 2000 and 2023.

In the attempt to find relevant studies in journals across different subjects—and research areas—a thorough and extensive search of seven databases was performed (53). To secure high recall without compromising precision, the following terms were included for block two about the phenomenon: “hope”, “hopeful”, “hopefulness”, “meaning making (process)”, “optimism”, “wish”, “wishful thinking” (see Supplementary Appendix S1 for the search in its entirety). Both “rehabilitation” and “recovery” were used as search terms for block three, as we wanted to include studies performed in an inpatient, as well as in an outpatient setting. Recovery is often linked to hope, thus a relevant term to include. The search terms and keywords were modified with input from an experienced librarian. Searches of EBSCO databases incorporating CINAHL, MEDLINE, and PsycINFO were undertaken together with SocINDEX, Social Work Abstracts, Eric and Web of Science (for further considerations on the choice of databases and the final search strategy see Supplementary Appendix S2). Any relevant protocol papers or conference abstracts identified from the database searches were followed up. A manual search of reference lists of relevant studies was also performed to complement the database search (53, 54).

#### 2.1.1 Study selection

Search results from the seven databases were combined in the bibliographic software RefWorks and duplicates removed. The primary aim was to identify studies exploring the perspective of persons with ABI, where the phenomenon of interest was hope. From initial pilot searches, it quickly became clear that the inclusion criteria had to be adjusted if the review was to include more than five or six studies. Thus, inclusion criteria were revised so that studies were included if they had hope as a substantial part of their findings even if hope had not been the initial focus of the study. Although this final search strategy also generated a significant number of irrelevant hits, it was seen as a necessary process to ensure that appropriate literature was captured.

#### 2.1.2 Eligibility criteria

The inclusion and exclusion criteria shown in Table 2 were used to determine eligible papers for study inclusion.

**Table 2:** Inclusion and exclusion criteria for study selection.

Inclusion criteria	Exclusion criteria
✓ Peer-reviewed academic journal articles	✓ Progressive diseases, terminal diseases, spinal cord injuries or concussion/mTBI.
✓ Adults with ABI^3^ (the age of 18 and above)	✓ Studies focusing only on the experiences of other than the person with ABI (e.g., rehabilitation professionals or family members).
✓ Studies that explored experiences of hope from firsthand perspective (the person with ABI).	✓ The effect of different interventions on hope
✓ Adults with ABI in a rehabilitation or recovery process.	✓ Acute phase, e.g., neurotrauma unit or ICY.
✓ Papers using qualitative methods.	✓ Grey literature, discussion papers, position papers, editorials, and commentaries.
✓ Published in English or Scandinavian language.	✓ Articles that did not present original material.
✓ Published between 2000 and 2023	

The selection of studies followed a two-stage process of screening and final selection. In order to ensure, systematicity and transparency the software Covidence was used in this process. Stage 1 (Screening): Titles and abstracts were screened against the inclusion and exclusion criteria and items deemed ineligible were removed. Stage 2 (Final selection): When articles appeared possibly to fit the inclusion criteria, full text copies of the articles were obtained. If it was not possible to determine the relevance by the title or abstract, a full text was also obtained to confirm or deny eligibility for inclusion (55). Full-text articles were then screened to ensure they met the inclusion and exclusion criteria and were relevant to the research question. Moreover, reference lists of the included studies were screened for further relevant studies.

#### 2.1.3 Quality assessment

After having completed full-text readings of the final studies, the Critical Appraisal Skills Program (CASP), a checklist for qualitative research, was used for the evaluation of the ten studies. The CASP toolkit consists of 10 questions to facilitate rapid evaluation (56). All articles were determined to be of good quality and although some studies were small scale studies, they could potentially be a of valuable contribution to understanding the experience of hope. Full details of the CASP quality appraisal are provided in Table 3.

**Table 3:** CASP quality appraisal for all ten articles.

CASP qualitative research checklist											
	Q1	Q2	Q3	Q4	Q5	Q6	Q7	Q8	Q9	Q10	% of Y
Alaszewski and Wilkinson (57)	Y	Y	Y	Y	Y	Y	Y	Y	Y	Positive	100&
Antelius (58)	Y	Y	Y	Y	Y	Y	N	Can’t tell	Y	Positive	90%
Bellon et al., (59)	Y	Y	Y	Y	Y	Y	Y	Y	Y	Positive	100%
Bright et al., (60)	Y	Y	Y	Y	Y	Y	Y	Y	Y	Positive	100%
Bright et al. (61)	Y	Y	Y	Y	Y	Y	Y/Can’t tell	Y	Y	Positive	90–100%
Lawton et al., (62)	Y	Y	Y	Y	Y	Y	Y	Y	Y	Positive	100%
Mairami et al. (63)	Y	Y	Y	Y	Y	Y	Y	Y	Y	Positive in regard to spirituality and faith, not much else	100%
Strong et al., (64)	Y	Y	Y	Y	Y	Y	Y	Y	Y	Positive, even though hope was a small part of the findings	100%
Taule et al., 2015 (65)	Y	Y	Y	Y	Y	Y	Y	Y	Y	Positive	100%
Tutton et al., 2012 (66)	Y	Y	Y	Y	Y	Y	Y	Y	Y	Positive	100%

1. Was there a clear statement of the aims of the research?

2. Is a qualitative methodology appropriate?

3. Was the research design appropriate to address the aims of the research?

4. Was the recruitment strategy appropriate to the aims of the research?

5. Was the data collected in a way that addressed the research issue?

6. Has the relationship between researcher and participants been adequately considered?

7. Have ethical issues been taken into consideration?

8. Was the data analysis sufficiently rigorous?

9. Is there a clear statement of findings?

10. How valuable is the research?

#### 2.1.4 Data extraction and synthesis methodology

The included studies were categorized according to their primary focus. For each study, the following data were obtained and put into the result table: Author & year, aim (of study), time since injury, sample (size, sex, and population), methods (e.g., type of qualitative study), data analysis and results.

To syntizese findings, the thematic synthesis methodology developed by Thomas and Harden was used (51). The approach outlines a three-step process to guide the thematic synthesis. It involves (1) close reading of text to identify data-driven patterns that become “categories for analysis” (67). (2) Next, codes are developed inductively using the results sections from the first article and transfere these to the succeeding articles after which new codes are added. Based on this process, authors then develop descriptive themes representative of groups of identified codes, that align closely to the literature being synthesized. The last step of the process is where (3) authors develop analytical themes which require them to apply their own interpretation of article findings (51). Both authors (51) read the articles multiple times. The initial reading was performed to develop an understanding of the topic and to get an overview on how each study met the purpose of this review. First author did the initial coding and extracted information about hope in the article findings, leaving out sections/themes that did not deal with this topic. Afterwards both authors discussed all the initial codes. Finally the first author established broader descriptive themes based on codes and with confirmed consistency of the ascribed text. Descriptive themes were put into a matrix and organized into analytical themes which were discussed by the authors to ensure agreement.

## 3 Results

**Figure 1:**
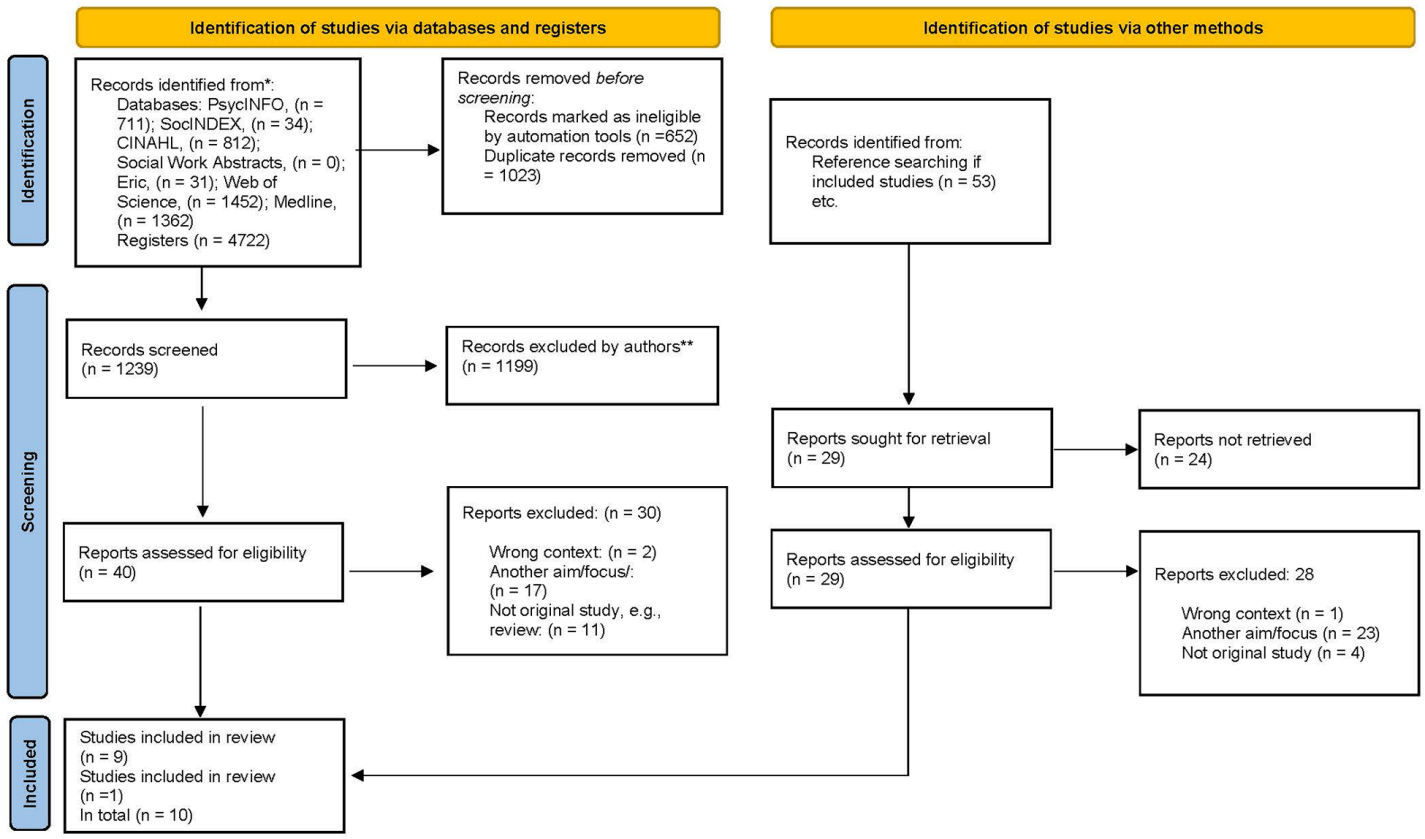
Prisma flow diagram.

Figure 1 provides the results of the database search in total within a Preferred Reporting Items for Systematic Reviews and Meta-Analyses (PRISMA 2020) flow diagram (50). A total of 4,722 abstracts were identified. After the removal of duplicate records and records marked as ineligible by automation tools, 1,239 abstracts were left. Following review of titles and abstracts, 40 publications were identified for potential inclusion. The full texts of these were read and assessed for eligibility.

Of the 40 studies retrieved for full text reading, only nine studies met the inclusion criteria. Hand-searching of the included articles' reference lists was also employed, where 29 studies were retrieved for full text reading, but only one additional study was identified which made a total of ten. The characteristics of these ten studies are presented in Table 4.

**Table 4:** Characteristics of included studies.

Study	Aim	Time since injury	Sample	Methods	Data analysis	Results
Alaszewski and Wilkinson (57) UK	To explore the experiences of hope seen from the perspective of stroke survivors under 60 years	3 months post injury and over an 18month period	Age: 21–60 *N* = 33 individuals with stroke (28 m, 15 f)	Interviews and diary entries.	Thematic content analysis	Hope was experienced as a deeply paradoxical and risk-laden notion (source of despair). The very idea of hope for the future was met as an unwelcome distraction and in some cases even as a source of distress.
Antelius (58) Sweden	To explore and deepen our understanding of hope in relation to severe disability and professionals’ involvement in narrating about the person and who this person is and will be, thus also a negotiation of hope.	Post rehabilitation and in chronic stage.	Age: 28–64, *N* = 7 professionals and *N* = 5 individuals with ABI.	Primarily interviews, but also participant observation, field notes and video recordings.	Narrative analysis of two adults with ABI in where the phenomenon of hope is explored. These narratives are coupled with the interview with professionals.	The professionals create narratives about the participants’ lives who the participants are and foremost who they will be, and how this narration must be understood in relation to both time and hope but also to training and motivation.
Bellon et al. (59) Australia	To examine the role of hope from the perspective of people living with traumatic and non-traumatic injuries.	Time since injury ranged from one to 57 years, (a median of 14 years).	Age: 28–65 *N* = 15 individuals with ABI (7 m, 8 f).	Semi-structured interviews	Thematic analysis	Five themes were found: the importance of hope, changes in hope over time, the connection between hope and positivity, factors influencing hope and the importance of fostering hope. Participants reported varying experiences, with increased levels of hope influenced by faith; supportive networks and professionals; goals; experiencing progress; having something to look forward to; and seeing others achieve. Professional attitudes were reported to either foster or negatively impact hope after brain injury.
Bright, Kayes, McCann and McPherson, 2013 (60) New Zealand	The aim of the study was to explore how hope was experienced by people with aphasia following stroke to identify factors influencing the experience of hope.	Time since injury ranged from two to five months.	Age:16–64 *N* = 5 individuals with stroke and aphasia (3 m., 2 f).	Semi-structured interviews	Narrative construction, thematic analysis, diagramming and memoring.	Participants appeared to experience hope in two ways: - Simply “having” hope (primary form of hope experienced by all participants) - Actively hoping (participants engaged with a future-oriented hope by identifying hopes for the future and working toward hopes) - Hope appeared dynamic, fluid and complex influenced by several variables.
Bright et al. (61) New Zealand	To identify how people with aphasia experience hope 1 year after stroke and how hope may change in the year after stroke.	Time since injury ranged from 14 to 18 months.	Age: 42–63 *N* = 4 individuals with stroke and aphasia (2 m, 2 f).	Interviews	Conventional content analysis and constant comparison.	Hope, identity, and social connectedness were closely entwined and could enable people to both dwell in the present and more towards desirable futures. Hope changed over time after stroke. It could take different forms: a broad sense of hope and an active hope for new possibilities in the future. Hoping for new possibilities appeared particularly vulnerable to a sense of a lack of progress, social isolation, and a loss of personal identity.
Lawton et al. (62) UK	To investigate people with aphasias’ subjective experiences and reflections of constructing and maintaining therapeutic alliances in aphasia rehabilitation.	All participants had been seen by more than one therapist for varying lengths of time (1–72 months).	Age: 45–88 *N* = 18 individuals with stroke and aphasia (12 m, 6 f).	In-depth interviews	Thematic content analysis	The emergent findings suggest that the establishment of a positive alliance not only impacted on engagement but also directly affects hope. Both instilling a sense of hope and maintaining hope were perceived to be by-products of a positive alliance. Instilling and preserving hope were interwoven into alliance development, through developing relational proximity, managing expectations sensitively and being attuned to individuals. Participants in this study wanted honest feedback about their recovery, which contrasts with earlier research suggesting some patients may wish to prioritise hope over realism.
Mairami et al. (63) Malaysia	To explore the contextual factors that mediate recovery following stroke in Malaysia, from the perspective of stroke survivors.	≤3: 12 4–10 year: 9 10 + years: 6	Age: 37–83 *N* = 27 individuals with stroke (21 m, 6 f). 5 of the participants had communication difficulties	In-depth interviews and observations	Thematic content analysis	Hope allowed individuals to have a positive mindset towards recovery. Several contextual factors influenced the recovery process. These factors, internal and external, exist interactively in the lived experiences of the survivors. Recovery was a personal journey that involved motivation, commitment, hard work and time and was therefore individually defined and experienced. Results suggest that the dynamics of the recovery process in stroke involves factors operating both within the individual and in interaction with the broader environment which should be considered. Hope and optimism, coping strategies, motivation and support from family and friends, and the use of alternative and complementary medicine shaped the process of recovery within a context where infra structure is extremely limited.
Strong et al. (64) USA	The purpose of this study was to examine the experiences of persons who had engaged in a project to co-construct personal narratives about life with aphasia.	Four-five years post injury.	Age: 55–66 *N* = 3 individuals with aphasia (3 m).	Interviews	Interpretative phenomenological analysis (IPA).	Three superordinate themes of these participants’ experience of the narrative co-construction process were identified and examined: (a) More than a story: It changed my life (b) A positive experience (c) Hope engendered by the co-construction experience empowered participants with new levels of confidence not only in their communication skills but also in themselves. The co-construction process allowed participants to express continuity with their existing identities through narrative.
Taule et al. (65) Norway	To explore mild-to moderate stroke survivors’ experiences with home rehabilitation after early supported discharge from hospital	6–8 months post injury.	Age: 45–80, *N* = 8 individuals with stroke (4 m, 4 f).	Interpretive interview design was used in the context of a randomized controlled trial.	Interpretive description, systematic text condensation and coping theory.	The core theme: Hope for a life worth living. Findings illustrate how vulnerable the participants’ hope is in encounters with professionals, and thus contribute to an ongoing discussion of how healthcare professionals can motivate patients and still facilitate a realistic view of recovery without severely upsetting them. Moreover, the results reveal how professionals’ responsibility to treat each patient using an individualised approach and supports that a closer cooperation between stakeholders and an expanded flexibility of the service might enhance this approach and build mutual confidence.
Tutton Seers et al. (66) UK	To explore the experience of hope for patients and staff in the context of a British stroke unit.	In the sub-acute phase after injury.	Age: 37–72, *N* = 10 individuals with stroke (3 f and 7 m). *N* = 10 multidisciplinary professionals	In-depth qualitative interviews	Ethnographic field notes and unstructured interviews	The findings identified the following themes: suffering, struggling with no hope and despair, hope for recovery and realistic hopefulness. Suffering and loss is inherent in the stroke experience and it is crucial that patients have the opportunity to express their feelings about hope and despair to be able to reframe their hope for the future. Emotional support over time, to develop the skills to create a supportive hopeful environment for patients is essential to maintain staff's own feelings of hopefulness.

### 3.1 Characteristics of the included studies

As shown in Table 4, half of the studies were carried out in the UK, Norway and Sweden (57, 58, 62, 65, 66) two in New Zealand (60, 61), and the rest in the USA (64), Australia (59), and Malaysia (63). The numbers of participants across studies varied considerably from three (64) to 33 participants (57). The age of the persons with ABI varied from 21 to 88 years old.

Six of the studies (59–62, 64, 65) used semi-structured or in-depth interviews to collect data whereas four of the studies collected data through a wider range of ethnographic approaches (57, 58, 63, 66) such as observations, diary entries, and video recordings. While eight of the studies focused on the experience of the person with an ABI, two examined the perspective of both the person with the ABI and of the professionals (58, 66). In these cases, only the persons with acquired brain injury's experiences of hope were included. All ten studies tended to include both female and male participants, but with a preponderance of male participants (46 females and 87 males).

The ten included studies in this review support the claim that most research on the phenomenon of hope has been conducted within the stroke population. Eight studies had persons with stroke as their population of interest, leaving only two on ABI as a broad population, including both people with stroke *and* traumatic brain injuries etc. (58, 59). A total of four studies focused specifically on persons with stroke and aphasia (60–62, 64).

Most studies were performed in the post-acute to chronic phase. Across studies, time since injury ranged from 2 months to 57 years. Bellon et al. and Antelius focused on the chronic phase with Bellon having participants ranging from one to 57 years since injury (median age of 14 years) (59), and Antelius exploring the understanding of hope through fieldwork in the context of a daily activity center rather than a rehabilitation center (58). On the other hand, Bright et al. (60) and Tutton et al. (66) focused on the post-acute phase, namely outpatient community-based rehabilitation at two to four months after injury at a regional acute stroke unit.

The qualitative studies revealed hope to be a very fluid and paradoxical notion. However, at the same time also something of great importance for the persons' rehabilitation or recovery process. This will be unfolded in the thematic synthesis of findings.

### 3.2 Thematic synthesis

Four analytical themes and eleven sub-themes were identified in the thematic synthesis and are shown in Table 5. Only two out of ten studies included persons with ABI (e.g., stroke and TBI etc.) thereby having a primary interest in the broad population of people with ABI. In all four analytical themes, studies with participants with TBI as well as participants with stroke are represented. Thus, themes were identified across the broad ABI population. The four analytical themes being: (1) hope a two folded phenomenon; (2) time and temporality; (3) progress, goals, and visibility; and (4) the alliance; a balancing act requiring good communication skills. The themes are described in the following section and selected quotations from the reviewed studies are used to support the analysis.

**Table 5:** The four analytical themes and sub-themes, with examples of quotes.

Analytical themes		Sub-themes		
1.	Hope a two folded phenomenon	*Hope as a driving force* I think if you haven’t got hope … you might as well die, yep*”* (59)*.*	*Hope as a source of despair* “You can hope for lots of stuff but then half the time it's going to disappear” (60)	* *
2.	Time & temporality	*Living one day at a time* Adrienne reported feeling “terrified” about the future which meant that she tried not to think about it, instead taking things “a day at a time”. …“just getting through” (60).	*A fixed future in later stages of the rehabilitation process?* I: “… Do you still want to be that (a Chef)”? Paul:”Aa…” I: “Do you think it's possible”? Paul:”No” (58).	*Self, identity and desirable futures* “I sort of thought ‘Oh, maybe. Maybe it's me’ and then I then I I thought about it you know, again the other night and I thought ‘Oh nah, it's not me’ and I just, I just I just can’t agree whether it's me or not” (60). “You know. I know now there's hope. I’m going to make it you know. I’ll be okay…” (64).
3.	Progress, goals, and visibility	*Progress intwined with hope and facilitated by rehabilitation professionals and peers* “She could see something in me, that yet although I could see it myself that was the goal I was going for. She could see beyond that, and she was getting me, by the scruff of the neck, that's where you want to be across there like … it just gave me enough kick up the pants to you know come back and fight” (62).	*Goals to make progress visible* “Keeping myself driven, I am good with that, that is very forward moving, having something to focus on giving me goals, targets, aims and pointers and stuff and that suits me…” (66).	*Goals based on personal wishes* “Sometimes she would lead the sessions and sometimes she would … not … … erm … … well … she would let me … have the floor and that, I think that was important as well” (62).
4.	The alliance, a balancing act requiring good communication skills	*Hope promoting relationships*	*Keeping hope on a leash* “Definitely, it is most positive for them [professionals] to … encourage people to have hope. As I said I’ve seen a lot of people who’ve given up the ghost, and a lot of times they’ve been … given bad advice or no hope by their so-called specialists, and they’ve given up” (59). “… they’re the ones that are offering hope”, or if they should be in his “no hope basket” (61).	*Professional competences* “She helped me because she understood uhh everything really so she umm helped me with me moods and things like that and family and understanding about you know because it's diffi it's diffi it's like a total change from what it was” (62).

#### 3.2.1 Theme 1: hope a two folded phenomenon

Hope turned out to be a very paradoxical phenomenon in which both a positive and negative side was embedded. Two sub-themes were identified in hope as a phenomenon.

##### 3.2.1.1 Sub-theme: hope as a driving force

Four of the studies identified hope as having a protective effect, supporting persons with ABI to continue and not give up (59, 60, 65, 66). Taule et al. went so far as to describe hope as the most important driving force in the person's struggle to make their situation comprehensible and worthwhile (65). According to Tutton et al., there was a real contrast between persons with stroke who were highly motivated and determined and for whom hope came easily, and those that felt low, lacking motivation or “flat with dense weakness and apparently had no hope” (66). As described by one of the participants with ABI in the study by Bellon: “I think if you haven’t got hope … you might as well die, yep” (59).

Two of the studies identified the fact that hope became something that allowed persons with ABI to have a positive mindset (59, 63). Through interviews, Mairami et al. identified that hope could originate either from within the person or from those around them, fostering optimism for a functional life (63). Factors identified by Bellon et al. as “drivers” for maintaining or increasing hope were, among other things:

• support
• having something to look forward to
• mental health (59).

For people with communication difficulties such as aphasia, the most common source of hope was social relations (other people) as well as spiritual beliefs (60). The meaning of faith and spiritual experiences was also mentioned in other studies. Mairami found that some people who were not spiritual or religious before the stroke turned to faith after their injury. This faith provided them with solace and hope for recovery (63). Similarly, persons with ABI in the study by Bellon et al. felt that faith was an important factor influencing hope: “I have faith and that gives me hope” (59).

##### 3.2.1.2 Sub-theme: hope as a source of despair

A connection between unfulfilled hopes and despair or emotional distress was identified in several studies, making it the flip side of hope (57, 59, 60, 65, 66). Persons with stroke experienced how hope could become a source of distress when it was connected to thoughts about the future (57). For persons with aphasia, hope was generally experienced as a good thing, but at the same time also difficult, since it could go unfulfilled (60). Uncertainty combined with thoughts about the future and having to redefine it, seemed to make persons with stroke vulnerable to feelings of distress and despair. Also, if the person had previous experiences of things gone wrong, they could be afraid that this would happen again and thus afraid to hope: “You can hope for lots of stuff but then half the time it's going to disappear” (60).

In the study by Bellon et al. some of the persons with ABI said that it could be difficult to gain hope and that hope was not always there:

“I guess I put hope in the same place I put happiness. Because … you’re always wanting to achieve it, or you’re always wanting to be in that state, but is that a realistic possibility? I don’t know” (59).

In a similar vain, one study described feelings of despair that could undermine hope and risk individuals falling into depression (66). Taken as a whole, the results indicate that hope is a multifaceted concept which can also have a negative side.

#### 3.2.2 Theme 2: time and temporality

Three of the studies included in this review illuminated the temporality connected to hope and hoping (58, 60, 61). Three sub-themes were identified in relation to this analytical theme.

##### 3.2.2.1 Sub-theme: living one day at a time

A present form of hope was identified as being dominant in people with stroke and aphasia. “Living in the moment” or “living one day at a time” was for many a response to the uncertainty inherent in thinking of the future—in effect, a way *not* to think about the future and to deal with the vulnerability that surfaced (60). In another study looking at how people with aphasia experienced hope one year post stroke and how it may have changed in that year (61), it was illustrated that the sense of hope was entwined with persons' views of their past and their present. Their perception of their poststroke recovery, well-being and view of the future was crucial in fostering—or threatening—their sense of hope for the future. Interestingly, Bright et al.'s study from 2020 suggested that the reference point for past comparisons shifted over time: in the post-acute phase, the comparisons were with their pre-stroke experience, whereas in the chronic phase they were with their immediate post-stroke experience (61). This could be seen as representing a process in which the persons revisited their hopes for the future and adjusted these based on their sense of what was possible and desirable: “I don’t believe hope can be the same [over time] as you [are not] the same” (61).

Bright et al. considered this movement in time to reflect the individual's desire, readiness, and capacity to move forward in life, actively constructing a future that they found both meaningful and imaginable (61).

##### 3.2.2.2 Sub-theme: a fixed future in later stages of the rehabilitation process

The concept of time and temporality was amplified when people were in later stages of their rehabilitation or recovery process, especially regarding the present. In a study in persons with severe brain injuries at a day center in Sweden, the future of the persons (in this stage of rehabilitation) was characterized as less open and limited in possibilities (58). In the quote below, a young seemed to have a less positive view of impressionable possibilities:

Interviewer (I): “If you hadn’t been in that car accident, what do you think would have happened then”?

Paul (P): “Chef. Chef”.

I: “Chef? Right, you were going to train to be a chef”?

P: “I was”

I: “You were in chef training? [P nods.] In high school”?

P: “KOMVUX” [municipal adult education].

[…]

I: “Do you still want to be that?”

P: “Aa”

I: “Do you think it's possible”?

P: “No” (58).

To him the horizon seemed rather distant as the future did not hold the possibility of becoming a chef again (58).

Persons with ABI resided in the present because they would never fully recover. Some of the persons with ABI talked about a fragile time horizon, dependent on their physical abilities in the future, and located their hope in their ability in the present to maintain their functional level (58). In temporal terms, you might therefore say that the horizon was further away than in the earlier stages of the persons' rehabilitation or recovery process.

For persons with stroke and aphasia it was described that a sense of hope persisted in the light of challenges, and the hope of things could be better was pivotal to getting through each day (61). By contrast, actively engaging in a future-oriented hope was identified by Bright et al. as quite troublesome for people with stroke and aphasia (60). Nevertheless, Strong et al. suggested that participation in a co-construction process^2^ with a speech-language pathologist was supporting persons with aphasia to engage in an intentional exploration of this process of actively hoping (64) making it into a sort of bridge and offering the possibility of getting the individual closer to that horizon.

##### 3.2.2.3 Sub-theme: self, identity, and desirable futures

Both studies that focused on self and identity centered on persons with stroke and aphasia (60, 64). It was experienced by persons in these studies that there was a disruption to their identity, leading to feelings of loss of identity. This loss of identity could also influence their experience of hope:

“I sort of thought ‘Oh, maybe. Maybe it's me’ and then I then I I thought about it you know, again the other night and I thought ‘Oh nah, it's not me’ and I just, I just I just can’t agree whether it's me or not” (60).

It was found that hope arose from what was perceived as meaningful to the participants and their sense of self, e.g., roles in life, sense of identity, faith, or an outlook on life. Hopes appeared to be relatively easy to identify and appeared from reflecting on past hopes and ways of being, the present and on their possible future (60). However, actively hoping could be more difficult for persons with communication difficulties as it was strongly language-based and involved identification of specific hopes. Hope, identity, and social connectedness were closely intertwined as hope could enable people with aphasia both to dwell in the present and to move towards to more desirable futures (61). The same study also found that persons with aphasia recalibrated their hopes for the future. This calibration process was influenced by the person's corresponding journey of identity re and co-construction. These two factors were therefore identified as significant in the recalibration of hope (61).

Strong et al. also found that the process of co-constructing a personal narrative with persons with stroke and aphasia was experienced as being positive (64). One of the individuals in this study felt it changed his life, suggesting that this process supported a positive view of identity. Hope engendered by the co-construction experience seemed to empower persons with stroke and aphasia with new levels of confidence, not only in their communication skills but also in themselves:

“You know. I know now there's hope. I’m I’m going to make it you know. I’ll be okay. I’ll get better and better. And and take time, but I’m getting better and better. And I I can do it. You know? I can I can I can do anything” (64).

As described earlier, Antelius stated in her study that: “In temporal terms there might be a horizon far away, but in narrative terms it is not an open one” (58). Applying a co-constructing intervention, as Strong et al. did in their study, seems to be a process that supports persons with communication difficulties (aphasia) to engage in an intentional exploration of the process of actively hoping (64), thus having the potential to open the horizon in narrative terms as well.

#### 3.2.3 Theme 3: progress, goals and visibility

Four of the included studies dealt specifically with progress and its significance in relation to hope (59–62). Three sub-themes were identified.

##### 3.2.3.1 Subtheme: progress intwined with hope and facilitated by rehabilitation professionals and peers

Seeing one's own progress as well as the progress of others was something that persons with stroke found to be a source of hope. Hope and progress appeared to have a mutually reinforcing effect (60, 61, 63).

For persons with aphasia, meeting other persons with aphasia was found to be important and instilled hope. Many had never heard of aphasia before, just as they had never met someone with aphasia, making it impossible for them to know what kind of progress was possible (60). In another study four persons were interviewed one year after their stroke, focusing on how hope might have changed during that year. The interviewees described increased hope for the future when they perceived they were making progress; conversely, a lack of progress threatened their sense of hope (60).

One study investigated the subjective experiences and reflections of persons with communication difficulties (aphasia) about constructing and maintaining therapeutic alliances in rehabilitation (62). In this process, the relationship between the person with aphasia and the health professional was shown to be of great importance. Even though this study focused on a specific group of professionals (speech and language therapists), the same may be true for other rehabilitation professionals. When progress was perceived as slow or imperceptible by the person, feedback from the therapist was of great importance as it was found to inculcate confidence and hope (62). As Terry in Lawton et al.'s study put it:

“[…] She could see something in me, that, yet although I could see it myself that was the goal I was going for. She could see beyond that, and she was getting me, by the scruff of the neck [gesturing], that's where you want to be across there like…it just gave me enough kick up the pants [laughs] to you know come back and fight” (62).

Thus, the therapist played a pivotal role, not only in making progress visible, but also in believing in the person, offering them hope for future improvement. It was precisely this kind of progress which was identified by persons with ABI as important in the maintenance and growth of hope (59). When persons with ABI in the study by Bellon et al. were asked to reflect on times where they felt they had made functional improvements or progress, they were thinking of milestones such as getting rid of a walking stick or being able to read a book in bed. Thus, every day events which showed their improvement and gave hope (59). Thus, the rehabilitation team was important for the participant's ability to build hope for recovery:

“They (municipal health care team) really came and stayed here and did something. They showed faith in positive development and supported me in that. It's important to convey that recovery can still happen, although the progress is slow” (65).

##### 3.2.3.2 Sub-theme: goals to make progress visible

Across the included studies, goals were identified as another important theme regarding hope (60–62, 64, 66). As expressed by one of the persons with ABI:

“…One aspect that they’ve really got right is focusing on the goal setting. It makes people more positive, and it gives them hope for the future” (59).

Persons with ABI emphasized the importance of setting goals that were achievable and realistic and warned of the dangers of having unachievable goals: “If I set my goals too high, I fall into a, like my depression goes up, and I get all, it's not worth it. So, I do things little by little” (59).

In studies including persons with stroke (60, 62, 64, 66), goals were found to be of importance since hope for recovery reflected the determination to move forward or to meet aspirational goals:

“Keeping myself driven, I am good with that, that is very forward moving, having something to focus on giving me goals, targets, aims and pointers and stuff and that suits me…” (66).

A strong multidisciplinary approach to rehabilitation using tangible goals to direct recovery seemed important for persons with stroke, as they used goals as a way of being hopeful about the future:

“I want to get rid of the sexy stockings (compression stockings) by the end of this week, you have to walk 10 meters unaided before they go. So they are going this week. I have decided” (66).

##### 3.2.3.3 Sub-theme: goals based on personal wishes

Professionals could risk undermining the hopes of the persons with ABI, and thereby increasing hopelessness or resignation about their future, by clinging to unrealistic hopes. This made it crucial to support people in imagining future possibilities when lack of progress hit (61). According to Lawton et al., genuine collaboration was dependent on the therapist's ability to listen carefully to the person with aphasia's narrative if they wanted to generate goals concordant with the person's priorities and needs (62). Persons who felt that their therapist had attempted to incorporate their personal wishes felt highly engaged with the process, which directly impacted on their engagement in rehabilitation:

“Sometimes she would lead the sessions and sometimes she would … not … … erm … … well … she would let me … have the floor and that, I think that was important as well” (62).

On the other hand, persons with stroke could feel inadequately understood, if the rehabilitation was not based on their personal wishes:

“They just had a plan of returning me back to work. It was their goal. When they repeated that every time we met, then I started to cry I think, every time” (65).

Strong et al. also found that the process of co-constructing a personal narrative with persons with aphasia was experienced as being positive, offering an opportunity for the individual to actively contemplate the future and their own goals (64).

#### 3.2.4 Theme four: the alliance; a balancing act requiring good communication skills

The importance of the therapeutic alliance and rehabilitation professionals' skills is a theme in more than half of the included studies in this review, making it a substantial factor relating to hope in a rehabilitation or recovery process (59–62, 64, 65). This theme seemed to be even more decisive when it came to persons with stroke and communication difficulties (aphasia) (60–62, 64). Three sub-themes were identified in relation to this analytical theme.

##### 3.2.4.1 Sub-theme: hope promoting relationships

The professionals' ability to provide hope for persons with stroke and aphasia appeared to be influenced by the strength of the therapeutic relationship (60). At the same time, engagement in the therapeutic process was described as central to the establishment of a partnership between the person with stroke and the health professional. An effective alliance could promote adherence and instill hope whereas an ineffective alliance eroded hope (60, 65). If the professionals were perceived to be too distant, maintaining rigid and inflexible boundaries, it could directly affect persons' engagement in rehabilitation as this was incompatible with their needs (62). This was especially true in the early stages of rehabilitation, during which persons with ABI were highly vulnerable and therefore needed more than professional distance.

On the other hand, being too attached posed a risk to independence and encouraged overreliance (62). Close alliances were characterized as genuine, friendly, non-judgmental, caring, open and connected. In order to provide the necessary infrastructure for establishing positive therapeutic relationships, the therapist needed to base it on honesty, trust, and respect (62). The therapeutic alliance was challenged when the person with ABI experienced unmanageable pressure about what to do or manage, as the confidence in the professional then was challenged. Also, professionals’ lack of expertise could challenge the alliance as this constituted a source of frustration for the person with ABI, as could controversies developing between persons with ABI and professionals or among professionals (65).

##### 3.2.4.2 Sub-theme: keeping hope on a leash

In the study by Bellon et al. the persons with ABI had mixed experiences of how professionals had encouraged or discouraged their hope, some recalling positive and some negative experiences. Negative experiences could involve the professionals being negative about the(ir) future (59). As a participant in the study by Bellon et al. explained:

“Definitely, it is most positive for them [professionals] to … encourage people to have hope. As I said I’ve seen a lot of people who’ve given up the ghost, and a lot of times they’ve been … given bad advice or no hope by their so-called specialists, and they’ve given up” (59).

Receiving a bleak prognosis or meeting professionals that were negative about their future had a negative impact on the person with ABI's experiences of hope.

Some persons with ABI felt that the therapists had a responsibility to mask their lack of faith in their progress. However, they also requested a balance between optimism and realism (65). Some even expressed that hope and encouragement from others were essential for them. Like in the case of Matthew in Bright et al.'s study (2020), who evaluated people for their hope giving qualities considering whether: “… they’re the ones that are offering hope”, or if they should be in his “no hope basket” (61).

##### 3.2.4.3 Sub-theme: professional competences

The skill set of professionals was of considerable importance across studies as professionals had the potential to discourage or encourage hope in the person with ABI (59, 60, 62, 65). According to Bellon et al., professionals could beneficially impact upon a person's experience of hope through person-centered and supportive therapeutic relationships (59). Taule and colleagues identified that successful encounters involving encouragement, empathy and equality seemed to promote the person with stroke's confidence in the professional's ability to help them manage their condition, as well as their sense of empowerment and their hope that they would be able to cope successfully in the future (65).

Participants appreciated and expressed a desire to cooperate with professionals who displayed essential therapeutic qualities, described as follows:

• Empathetic
• Taking time to listen
• Offering comfort
• Being nice
• Enthusiastic
• Making optimistic statements (65).

One skill that persons with stroke and aphasia identified as particularly important in helping them to think positive and be hopeful about the future was the professionals’ ability to be supportive. It was also perceived as necessary for the therapist to have highly attuned communication skills in order for the person with aphasia to have a sense of being heard and understood (60, 62). Skills like empathetic understanding (not just understanding), giving time and getting to know the human being (not just the diagnosis) were potentially of even greater importance if the persons were to avoid isolation, build trust and maintain hope. As the participant in the study by Lawton expressed it:

“She (professional) helped me because she understood uhh everything really so she umm helped me with me moods and things like that and family and understanding about you know because it's diffi it's diffi it's like a total change from what it was” (62).

## 4 Discussion

### 4.1 Summary of main findings

This qualitative synthesis of evidence highlights several key issues for persons with ABI to sustain and instill hope in a time of uncertainty and hopelessness. The four analytical themes and eleven sub-themes identified through thematic synthesis were (1) hope a two folded phenomenon, with the sub-themes “hope as a driving force” and “hope as a source of despair”; (2) time and temporality, with the sub-themes “living one day at a time”, “a fixed future in later stages of the rehabilitation process?” and “self, identity and desirable futures”; (3) progress, goals and visibility, with the sub-themes “progress intwined with hope and facilitated by rehabilitation professionals and peers”, “goals to make progress visible”; and “goals based on personal wishes” and lastly (4) the alliance; a balancing act requiring good communication skills, with the sub-themes “hope promoting relationships”, “keeping hope on a leash” and “professional competences”.

Previous systematic qualitative reviews have primarily explored hope in persons with stroke and left out other subgroups of ABI such as people with traumatic brain injury, hemorrhages in the brain other than stroke etc. (36, 44, 45). Moreover, most of the included studies in these reviews did not give voice to people with communication difficulties following ABI as they were often excluded (ibid).

This review includes all ABI groups and the findings are in line with previous reviews, in that hope is multifaceted (potentially having a positive as well as a negative effect), influenced by several factors (e.g., personal, or social), and crucially related to the establishment of goals (36, 44, 45). All four analytical themes were identified based on experiences that included both persons with TBI, haemorrhage etc. as well as stroke.

Our review highlights several good reasons to pay attention to time and temporality. First, time and temporality serve as a frame within which everything else unfolds, including the rehabilitation or recovery process. Secondly, time and temporality are shown to be of great importance for persons with ABI, especially in the later stages of their rehabilitation or recovery process. The results suggest that hope is to be found elsewhere in the more chronic phases of the rehabilitation, calling for different perspectives and amplifying the importance of the present instead of having a fixed view of the future. For persons with communication difficulties, time and temporality seems to play into their necessity to be rooted in time—specifically, in the present, which serves as an anchor and makes it possible to look at the future with the support of rehabilitation professionals. This leaves us with the question of whether it is easier to maintain or instill hope in earlier stages of rehabilitation compared to later stages? A study by Bright et al. showed that conversations about the future also seems to be constrained and limited in the inpatient setting poststroke (68). The study explored how clinicians talk about the future with patients and Bright et al. found that these types of conversations were constrained to short-term futures and limited to what aspects of life after stroke were discussed. According to Bright et al. being able to create conversational and relational spaces where people are supported to look into the future with a sense of possibility, hope, and potential is vital for persons to move forward in their lives post stroke. Based on their findings they conclude that, communication must be seen as a core clinical skill and a clinical intervention in its own right (68).

Our findings suggests that the relationship to professionals can promote adherence and instill hope, especially for persons with aphasia. This is supported by a meta-synthesis from 2020, where relationships were identified as being critical for persons experiencing communication impairment after stroke (69). In practice, this requires strong communicative competences in rehabilitation professionals (70, 71).

Communication partner training is an approach that has shown to be useful in supporting communication skills (72–76). In Denmark the method Supported Conversation for Adult with Aphasia (SCA) is relatively widespread in practice. A review by Christensen et al. shows that also cognitive communication disorders of people with TBI challenge the interaction between rehabilitation professionals and persons with TBI. Their findings demonstrate that the communicative barrier is closely related to the professionals' communicative approach. Moreover, that rehabilitation professionals holding a collaborative and acknowledging approach using supportive strategies are the ones that may facilitate successful communicative interactions (77). This point to the fact that rehabilitation professionals must be given the opportunity to acquire the necessary competencies in communication strategies.

Rehabilitation strives for a coherent approach, with the bio-psycho-social model forming the basis for rehabilitation (32, 33). Danish research in the field of brain injury has nevertheless shown that rehabilitation continues to primarily be aimed at physical and functional rehabilitation and does not adopt a holistic focus where the person's existential and emotional situation is considered (32, 33). Rehabilitation of the person's “inner life world” (inside-perspective), understood as dealing and coping with personal, identity-related, and emotional challenges, including hope, is given less focus, although it is an important and central aspect of a bio-psycho-social approach (32, 33). Being a professionally skilled occupational therapist or neuropsychologist is therefore not enough. In addition professionals need personal competences (70). According to Deegan, who has lived experience with disabilities, a decisive step toward understanding the experience of the person with a disability is that rehabilitation professionals embrace and accept their own woundedness and vulnerability because in doing so, “we share a common humanity” (78).

According to the Mattingly, professionals often focus on what she calls “the medical hope”, a narrow understanding where hope alone is tied to curation and rehabilitation in a more traditional sense. The hope of persons with an illness or disability, however, often involves a much broader understanding of hope as a journey towards healing or recovery, trying to find a positive meaning in what has happened (79–83).

The biomedical model offers no definitive cure when it comes to the physical and psychological consequences of an ABI. However, recovery is not only a pathological matter. Regaining quality of life can happen despite pathological consequences. Thus, there is an imperative need to hope even when everything seems hopeless. Hopelessness can have major consequences for the person, as it has been shown to be associated with depression and suicide across diagnoses (84–86). Hope, on the other hand, has been shown to have a positive impact on rehabilitation outcomes both for persons with mental and physical impairments (32, 33, 84, 87, 88). Within neurorehabilitation, it has been seen as good practice to confront persons with ABI with their disabilities since this could lead to greater insight and acceptance of difficulties and thus to a greater motivation to explore their limitations. In practice, however, it appears that confrontations do not necessarily lead to increased motivation. On the contrary, confrontations can lead either to individuals giving up and thus losing motivation and hope, or to mental resistance (89). This leaves rehabilitation professionals to walk a thin line between encouraging and discouraging hope when it comes to especially persons with ABI lacking insight in their own impairments.

Our analysis furthermore reveals that hope, identity, and social connectedness are closely intertwined as hope gives persons with aphasia the possibility both to dwell in the present but also to move forward. The co-construction of identity change are significant in the person's ability to recalibrate hope, which is often necessary during a rehabilitation or recovery process. For years anthropologists have written about the positive traits of narratives, in that story—told or acted upon—carry a healing potential through their contribution to the transformation of identity, interpretation of the past and even the creation of future scenarios (82, 90). When persons acquire a brain injury and are faced with an uncertain future, focusing on life as lived in and over time can therefore make good sense, since such life-changing experiences create a natural focus on both the past and the future. Our review suggests that collaborating with the person on co-constructing a personal narrative holds the potential to support a positive view of identity and to empower persons with aphasia (or other communication difficulties) with new levels of confidence in themselves. This is in line with research within the field of communication disorders, where several studies talk about co-constructed communication as being essential in offering communication partners a way to approach everyday conversation and supporting positive narratives that reaffirm a sense of self (91–93).

Professionals may be afraid of giving false hope, which is why they may end up promoting too little hope to the person with ABI. Being with persons who have wishes, hopes, and dreams for the future, and who at the same time are in a situation that complicates the achievement of these, can be experienced as difficult for professionals. Findings in this review suggest that there is an opposite relationship between professionals playing a vital role, not only in making progress visible, but also in offering persons with ABI hope for future improvement.

Being the “holder” of another human being's hope is not an easy task. In fear of giving false hope, professionals may imply and maintain a less hopeful rehabilitation perspective instead (66). It appears that professionals’ capacity to contain despair and maintain hope prevents the person from giving up and feeling devastated by the brain injury. This require professional skills to tolerate the uncertainty and despair of the person's life. This kind of support is particularly crucial in times when progress is slow.

As we clearly see in this and other reviews, goals are connected to hope. In an ethnographic study by Tonnesen and Nielsen among persons with Parkinson's disease, they found that rehabilitation goals could appear as steppingstones towards hope (94). The correlation between goals and hope is also emphasized in this review. Progress seems to be paramount for the person's ability to maintain hope over time, thereby making goals a way for professionals to make progress visible. Researchers such as Levack and colleagues have also linked rehabilitation goals and hope. Levack et al. found that rehabilitation goals may keep hope alive by symbolizing possible progress (95). In a Cochrane review from 2008, Levack and colleagues concluded that there is low quality evidence that goal setting may improve outcomes for adults in rehabilitation with various disabilities, and moreover that the best graded evidence favors positive effects for psychosocial outcomes (such as emotional status, health-related quality of life, etc.) rather than physical outcomes (95). In this review, persons with ABI and professionals have a clear focus on goal setting in relation to their physical functions, whereas functions in relation to psychosocial conditions are absent. Once again, it is important to stress that goals relating to the psychosocial dimension are equally important when trying to uphold a bio-psycho-social approach in rehabilitation.

Future research should consider looking at experiences of hope in specific stages of the person's rehabilitation or recovery process. This could be of importance for further understanding of the concept of hope as it develops especially over time and in time. Furthermore, future research should consider exploring whether gender, age, social network or pre-injury personality influence the way in which persons with ABI experience hope in their rehabilitation or recovery process. Last, a specific focus on persons with communication difficulties—specifically the subgroup of persons with TBI and cognitive communication disorders, could be of great value as this is often an overlooked group. This group of persons may have unique needs that warrant separate exploration which is why it is important to also include studies that provide persons with communication difficulties with a voice (47).

### 4.2 Implications for practice

In light of our results, professionals are advised to consider the impact of giving or taking hope away when it comes to the population of persons with ABI. This review supports the need for practice to acknowledge the deep and fundamental importance of the concept of hope as a major part of the rehabilitation and recovery of persons with ABI, and even more so for persons with communication difficulties such as aphasia.

The literature included in this study provides an insight into why hope is often described as a paradox, having both positive and negative qualities embedded. This paradoxical concept requires professionals to make individual assessments of each person with ABI to find out how hope can best be supported, in the light of the person's personality, social network, etc., as well as where the person is in their rehabilitation or recovery process.

Especially for the subgroup of persons with communication difficulties, there seems to be a need for professionals to acquire skills in how to support conversation and the co-construction of narratives. This entails knowledge on how to work with the personal narratives of the individual, making it crucial that professionals gain qualifications on how to use supportive conversation techniques and tools.

At the same time, this review underlines that professionals play a crucial role when it comes to maintaining supportive relationships and instilling hope throughout the rehabilitation or recovery process. The role requires professionals to value the humanity and personhood of the persons, making them feel heard and understood and keeping a hopeful stance about their potential for recovery. This calls for highly attuned communication skills, along with the ability to contain despair and uncertainty on behalf of the person without losing hope entirely.

If practice therefore wants to offer rehabilitation grounded in a bio-psycho-social approach, then the psychosocial wellbeing of the person needs to be a primary outcome of the rehabilitation on an equal footing with outcomes concerning physical functions. Thus, professionals need to have a raised awareness of the emotional support required if an individual is to recover from a brain injury as a whole person. That is to refer to appropriate health professionals and have cross disciplinary collaborations. This again points to the necessity of setting goals for the physical and more practical part of the person's rehabilitation as well as the psychosocial part of the person's overall recovery.

Professionals should be knowledgeable about the emotional dimensions of rehabilitation during their training and education. The current professional training sequence includes limited information about the relational impact professionals can have on persons in rehabilitation and the psychosocial dimensions of this process. If we are to pay greater attention to social and psychological factors in the lives of people with disabilities and chronic health conditions, there need to be opportunities for cross-disciplinary professionals in general to have continuing education in psychosocial rehabilitation and to work with and develop their own personal skills at various levels of training.

### 4.3 Limitations

We have used an aggregative review form, namely a systematic review of qualitative research, as we wanted to accumulate knowledge relevant to the phenomenon of hope and show relationships among the pieces of knowledge across the included studies (96). In doing so we have tried to guide the selection of studies by clear eligibility criteria just as a systematic approach has been applied in the different steps of the review process. A review protocol was outlined and adapted along with the search process but only used as a working tool for the authors and therefore not registered at e.g., PROSPERO.

With this review we wanted to explore hope as experienced by the broad ABI population in a rehabilitation or recovery setting. This resulted in studies that cut across diagnostic groups covering many different impairments and taking place in a mixture of in- and outpatient services at several different stages of the rehabilitation or recovery process. In our thematic synthesis we have tried to be transparent about the different contexts of the included studies, but nevertheless the broad scope of this review is both a strength and a weakness. There is a limitation upon transferability of the findings in relation to the contextual data presented in the studies that could have been followed more closely throughout the review if the context of the studies had been more alike.

Although some of the included studies in this review only had few participants the data from these qualitative studies were of great importance as they gave valuable insight into an often-excluded group: People with ABI and communication difficulties (aphasia). So even though some would argue it to be a limitation (small sample size), it proved to be necessary in this qualitative review in order to represent the broad group of people with ABI.

Most of the included studies were gathered in high or middle-income countries which probably has a significance in that other perspectives could be imagined to be present in low-income countries, which possibly do not have the same infrastructure around healthcare.

The thematic synthesis methodology applied to this review offers a transparent approach to the synthesis of the primary studies; however, future research should consider whether other approaches used to analyse data could help bring other aspects of hope to the surface.

### 4.4 Conclusions

According to the findings of this review, rehabilitation professionals should embrace the ambiguity of hope, as a driving force as well as a source of despair. Attention must be paid to time and temporality and how to make progress visible as well as tangible for persons with ABI during their rehabilitation or recovery process. Finally, rehabilitation professionals must acknowledge the relationship with the individual with ABI as a core component of the rehabilitation in general. This requires a focus on professionals' personal competences, as a hope-promoting relationship between the professional and the person with ABI seems to be needed in order to accommodate to the psycho-social part of the bio-psycho-social approach.

## Footnotes

^1^PICo worksheet and search strategy. US National Center for Dental Hygiene Research. The form is accessible at KUB’s (Det kongelige bibliotek) website [Forskningsspørgsmål, PICO og andre metoder—Systematiske reviews—Index at The Royal Danish Library (kb.dk)].

^2^A co-construction process where the person with aphasia was supported in having their story written down (who I was before my stroke/aphasia, my stroke and aphasia, who I am today, my future/goals), potentially including support from a family member, for instance clarifying a specific point in the story, or the clinician, for instance uploading a photo.
