# Supplementary material for: Hope as experienced by people with acquired brain injury in a rehabilitation—or recovery process: a qualitative systematic review and thematic synthesis
Source: Front Rehabil Sci. 2024 May 14;5:1376895. doi: 10.3389/fresc.2024.1376895 (PMC11131419; doi:10.3389/fresc.2024.1376895)
Supplement: Supplementary file 2 [file Datasheet2.docx]

**Appendix 2; Search Protocol; Hope, Stroke & Rehabilitation**

**Number of hits for the various searches in the seven bases:**

| **Base** | **Block 1 (stroke) AND Block 2 (hope) AND block 3 (Rehabilitation)** | **Limited to Peer Reviewed, published between 2021-2023 in English** |
| --- | --- | --- |
| **PsycINFO** | 394 | 27 |
| **SocINDEX** | 22 | 2 |
| **CINAHL** | 446 | 31 |
| **Social Work Abstracts** | 0 | 0 |
| **ERIC** | 19 | 1 |
| **Web of Science** | 808 | 149 |
| **Medline** | 783 | 122 |
| **I alt:** | 2472 | 332 |
| **After removing duplicates:** | **107** | |

**Search in PsycINFO**

Search made the 24/01. 2023

**Block 1: Stroke**

| **Nr.** | **Search term** | **PsycINFO** | **Number of hits** |
| --- | --- | --- | --- |
| 1 | Stroke | TI stroke OR SU stroke OR AB stroke | 40,635 |
| 2 | Brain injury | TI “brain injur*” OR SU “brain injur*” OR AB “brain injur*” | 35,955 |
| 3 | Head injury | TI “head injur*” OR SU “head injur*” OR AB “head injur*” | 8,190 |
| 4 | Head trauma | TI “head trauma” OR SU “head trauma” OR AB “head trauma” | 2,043 |
| 5 | Traumatic brain injury | TI “traumatic brain injur*” OR SU “traumatic brain injur*” OR AB “traumatic brain injur*” | 24,411 |
| 6 | TBI | TI TBI OR SU TBI OR AB TBI | 12,909 |
| 7 | Brain trauma | TI “brain trauma” OR SU “brain trauma” OR AB “brain trauma” | 756 |
| 8 | Acquired brain injury | TI “acquired brain injur*” OR SU “acquired brain injur*” OR AB “acquired brain injur*” | 2,533 |
| 9 | ABI | TI ABI OR SU ABI OR AB ABI | 1,592 |
| 10 | Brain damage | TI “brain damage*” OR SU “brain damage*” OR AB “brain damage*” | 26,838 |
| 11 | Brain infarction | TI “brain infarction*” OR SU “brain infarction*” OR AB “brain infarction*” | 1,071 |
| 12 | Cerebral infarction | TI “cerebral infarction” OR SU “cerebral infarction” OR AB “cerebral infarction” | 3,014 |
| 13 | Apoplexia | TI apoplex* OR SU apoplex* OR AB apoplex* | 151 |
| 14 | Neurological impairment | TI “neurological impairment*” OR SU “neurological impairment*” OR AB “neurological impairment*” | 1,454 |
| 15 | Central nervous system neoplasm | TI “central nervous system neoplasm*” OR SU “central nervous system neoplasm*” OR AB “central nervous system neoplasm*” | 205 |
| 16 | Central nervous system tumor | TI “central nervous system tumor*” OR SU “central nervous system tumor*” OR AB “central nervous system tumor*” | 144 |
| 17 | Cerebral hemorrhage | TI “cerebral hemorrhage*” OR SU “cerebral hemorrhage*” OR AB “cerebral hemorrhage*” | 3,354 |
| 18 | Cerebrovascular accident | TI “cerebrovascular accident*” OR SU “cerebrovascular accident*” OR AB “cerebrovascular accident*” | 24,301 |
| 19 | Encephalopathy | TI encephalopath* OR SU encephalopath* OR AB encephalopath* | 7,232 |
| 20 | Subarachnoid hemorrhage | TI “subarachnoid hemorrhage*” OR SU “subarachnoid hemorrhage*” OR AB “subarachnoid hemorrhage*” | 1,694 |
| 21 |  | DE "Brain Injuries" | 12,472 |
| 22 |  | DE "Traumatic Brain Injury" | 20,675 |
| 23 |  | DE "Brain Damage" | 17,848 |
| 24 |  | DE "Cerebral Hemorrhage" | 3,141 |
| 25 |  | DE "Cerebrovascular Accidents" | 23,972 |
| 26 |  | DE "Encephalopathies" | 3,494 |
| 27 |  | DE "Subarachnoid Hemorrhage" | 1,229 |
| 28 |  | S1 OR S2 OR S3 OR S4 OR S5 OR S6 OR S7 OR S8 OR S9 OR S10 OR S11 OR S12 OR S13 OR S14 OR S15 OR S16 OR S17 OR S18 OR S19 OR S20 OR S21 OR S22 OR S23 OR S24 OR S25 OR S26 OR S27 | 109,234 |

**Block 2: Hope**

| **Nr.** | **Search term** | **PsycINFO** | **Number of hits** |
| --- | --- | --- | --- |
| 29 | Hope | TI hope OR SU hope OR AB hope | 48,755 |
| 30 | Hopeful | TI hopeful OR SU hopeful OR AB hopeful | 2,619 |
| 31 | Hopefulness | TI hopefulness OR SU hopefulness OR AB hopefulness | 731 |
| 32 | Hopelessness | TI hopelessness OR SU hopelessness OR AB hopelessness | 7,056 |
| 33 | Despair | TI despair OR SU despair OR AB despair | 3,990 |
| 34 | Meaning making | ( TI "meaning making" OR SU "meaning making" OR AB "meaning making" ) OR ( TI "meaning-making" OR SU "meaning-making" OR AB "meaning-making" ) | 5,838 |
| 35 | Meaning making process | ( TI "meaning making process*" OR SU "meaning making process*" OR AB "meaning making process*" ) OR ( TI "meaning-making process*" OR SU "meaning-making process*" OR AB "meaning-making process*" ) | 773 |
| 36 | Optimism | TI optimism OR SU optimism OR AB optimism | 12,507 |
| 37 | Pessimism | TI pessimism OR SU pessimism OR AB pessimism | 3,358 |
| 38 | Wish | TI wish OR SU wish OR AB wish | 26,433 |
| 39 | Wishful thinking | TI "wishful thinking" OR SU "wishful thinking" OR AB "wishful thinking" | 803 |
| 40 |  | DE "Hope" | 4,277 |
| 41 |  | DE "Hopelessness" | 2,207 |
| 42 |  | DE "Optimism" | 5,077 |
| 43 |  | DE "Pessimism" | 1,439 |
| 44 |  | DE "Wishful Thinking" | 181 |
| 45 |  | S29 OR S30 OR S31 OR S32 OR S33 OR S34 OR S35 OR S36 OR S37 OR S38 OR S39 OR S40 OR S41 OR S42 OR S43 OR S44 | 104,185 |

**Block 1 (Stroke) AND Block 2 (Hope):**

| 46 |  | S28 AND S45 | 1,182 |
| --- | --- | --- | --- |

**Block 3: Rehabilitation**

| **Nr.** | **Search term** | **PsycINFO** | **Number of hits** |
| --- | --- | --- | --- |
| 47 | Rehabilitation | TI rehabilitation OR SU rehabilitation OR AB rehabilitation | 77,818 |
| 48 | Rehabilitation process | TI “rehabilitation process*” OR SU “rehabilitation process*” OR AB “rehabilitation process*” | 1,599 |
| 49 | Rehabilitation journey | TI "rehabilitation journey” OR SU “rehabilitation journey” OR AB “rehabilitation journey” | 12 |
| 50 | Recovery | TI recovery OR SU recovery OR AB recovery | 80,704 |
| 51 | Recovery process | TI "recovery process*” OR SU "recovery process*” OR AB "recovery process*” | 3,267 |
| 52 | Recovery journey | TI "recovery journey” OR SU "recovery journey” OR AB "recovery journey” | 176 |
| 53 |  | DE "Rehabilitation" | 23,295 |
| 54 |  | DE "Recovery (Disorders)" | 14,583 |
| 55 |  | S47 OR S48 OR S49 OR S50 OR S51 OR S52 OR S53 OR S54 | 150,343 |

**Block 1 (Stroke) AND Block 2 (Hope) AND Block 3 (Rehabilitation):**

| 56 |  | S28 AND S45 AND S55 | 394 |
| --- | --- | --- | --- |

| **57** |  | **S28 AND S45 AND S55**  **Limiters - Date of Publication: 20210101-20231231**  **Narrow by Language: - english.** | **27** |
| --- | --- | --- | --- |

**Search in SocINDEX**

Search made the 24/01. 2023

**Block 1: Stroke**

| **Nr.** | **Search term** | **SocINDEX** | **Number of hits** |
| --- | --- | --- | --- |
| 1 | Stroke | TI stroke OR SU stroke OR AB stroke | 2,598 |
| 2 | Brain injury | TI “brain injur*” OR SU “brain injur*” OR AB “brain injur*” | 1,120 |
| 3 | Head injury | TI “head injur*” OR SU “head injur*” OR AB “head injur*” | 616 |
| 4 | Head trauma | TI “head trauma” OR SU “head trauma” OR AB “head trauma” | 210 |
| 5 | Traumatic brain injury | TI “traumatic brain injur*” OR SU “traumatic brain injur*” OR AB “traumatic brain injur*” | 554 |
| 6 | TBI | TI TBI OR SU TBI OR AB TBI | 317 |
| 7 | Brain trauma | TI “brain trauma” OR SU “brain trauma” OR AB “brain trauma” | 24 |
| 8 | Acquired brain injury | TI “acquired brain injur*” OR SU “acquired brain injur*” OR AB “acquired brain injur*” | 108 |
| 9 | ABI | TI ABI OR SU ABI OR AB ABI | 258 |
| 10 | Brain damage | TI “brain damage*” OR SU “brain damage*” OR AB “brain damage*” | 691 |
| 11 | Brain infarction | TI “brain infarction*” OR SU “brain infarction*” OR AB “brain infarction*” | 9 |
| 12 | Cerebral infarction | TI “cerebral infarction” OR SU “cerebral infarction” OR AB “cerebral infarction” | 27 |
| 13 | Apoplexia | TI apoplex* OR SU apoplex* OR AB apoplex* | 17 |
| 14 | Neurological impairment | TI “neurological impairment*” OR SU “neurological impairment*” OR AB “neurological impairment*” | 70 |
| 15 | Central nervous system neoplasm | TI “central nervous system neoplasm*” OR SU “central nervous system neoplasm*” OR AB “central nervous system neoplasm*” | 0 |
| 16 | Central nervous system tumor | TI “central nervous system tumor*” OR SU “central nervous system tumor*” OR AB “central nervous system tumor*” | 10 |
| 17 | Cerebral hemorrhage | TI “cerebral hemorrhage*” OR SU “cerebral hemorrhage*” OR AB “cerebral hemorrhage*” | 43 |
| 18 | Cerebrovascular accident | TI “cerebrovascular accident*” OR SU “cerebrovascular accident*” OR AB “cerebrovascular accident*” | 51 |
| 19 | Encephalopathy | TI encephalopath* OR SU encephalopath* OR AB encephalopath* | 296 |
| 20 | Subarachnoid hemorrhage | TI “subarachnoid hemorrhage*” OR SU “subarachnoid hemorrhage*” OR AB “subarachnoid hemorrhage*” | 40 |
| 21 |  | DE "STROKE" | 385 |
| 22 |  | S1 OR S2 OR S3 OR S4 OR S5 OR S6 OR S7 OR S8 OR S9 OR S10 OR S11 OR S12 OR S13 OR S14 OR S15 OR S16 OR S17 OR S18 OR S19 OR S20 OR S21 | 5,510 |

**Block 2: Hope**

| **Nr.** | **Search term** | **SocINDEX** | **Number of hits** |
| --- | --- | --- | --- |
| 23 | Hope | TI hope OR SU hope OR AB hope | 17,700 |
| 24 | Hopeful | TI hopeful OR SU hopeful OR AB hopeful | 982 |
| 25 | Hopefulness | TI hopefulness OR SU hopefulness OR AB hopefulness | 164 |
| 26 | Hopelessness | TI hopelessness OR SU hopelessness OR AB hopelessness | 1,139 |
| 27 | Despair | TI despair OR SU despair OR AB despair | 1,730 |
| 28 | Meaning making | ( TI "meaning making" OR SU "meaning making" OR AB "meaning making" ) OR ( TI "meaning-making" OR SU "meaning-making" OR AB "meaning-making" ) | 1,379 |
| 29 | Meaning making process | ( TI "meaning making process*" OR SU "meaning making process*" OR AB "meaning making process*" ) OR ( TI "meaning-making process*" OR SU "meaning-making process*" OR AB "meaning-making process*" ) | 174 |
| 30 | Optimism | TI optimism OR SU optimism OR AB optimism | 3,906 |
| 31 | Pessimism | TI pessimism OR SU pessimism OR AB pessimism | 1,338 |
| 32 | Wish | TI wish OR SU wish OR AB wish | 8,756 |
| 33 | Wishful thinking | TI "wishful thinking" OR SU "wishful thinking" OR AB "wishful thinking" | 225 |
| 34 |  | DE "HOPE" | 1,246 |
| 35 |  | DE "DESPAIR" | 504 |
| 36 |  | DE "OPTIMISM" | 1,241 |
| 37 |  | DE "PESSIMISM" | 498 |
| 38 |  | S23 OR S24 OR S25 OR S26 OR S27 OR S28 OR S29 OR S30 OR S31 OR S32 OR S33 OR S34 OR S35 OR S36 OR S37 | 35,075 |

**Block 1 (Stroke) AND Block 2 (Hope):**

| 39 |  | S22 AND S38 | 91 |
| --- | --- | --- | --- |

**Block 3: Rehabilitation**

| **Nr.** | **Search term** | **SocINDEX** | **Number of hits** |
| --- | --- | --- | --- |
| 40 | Rehabilitation | TI rehabilitation OR SU rehabilitation OR AB rehabilitation | 21,126 |
| 41 | Rehabilitation process | TI “rehabilitation process*” OR SU “rehabilitation process*” OR AB “rehabilitation process*” | 269 |
| 42 | Rehabilitation journey | TI "rehabilitation journey” OR SU “rehabilitation journey” OR AB “rehabilitation journey” | 0 |
| 43 | Recovery | TI recovery OR SU recovery OR AB recovery | 14,265 |
| 44 | Recovery process | TI "recovery process*” OR SU "recovery process*” OR AB "recovery process*” | 690 |
| 45 | Recovery journey | TI "recovery journey” OR SU "recovery journey” OR AB "recovery journey” | 17 |
| 46 |  | DE "REHABILITATION" | 4,967 |
| 47 |  | S40 OR S41 OR S42 OR S43 OR S44 OR S45 OR S46 | 34,668 |

**Block 1 (Stroke) AND Block 2 (Hope) AND Block 3 (Rehabilitation):**

| 48 |  | S22 AND S38 AND S47 | 22 |
| --- | --- | --- | --- |

| **49** |  | **S22 AND S38 AND S47**  **Limiters - Scholarly (Peer Reviewed) Journals; Date of Publication: 20211201-20231231**  **Narrow by Language: - English** | **2** |
| --- | --- | --- | --- |

**Search in CINAHL**

Search made the 24/01 2023

**Block 1: Stroke**

| **Nr.** | **Search term** | **CINAHL** | **Number of hits** |
| --- | --- | --- | --- |
| 1 | Stroke | TI stroke OR SU stroke OR AB stroke | 141,095 |
| 2 | Brain injury | TI “brain injur*” OR SU “brain injur*” OR AB “brain injur*” | 38,020 |
| 3 | Head injury | TI “head injur*” OR SU “head injur*” OR AB “head injur*” | 11,838 |
| 4 | Head trauma | TI “head trauma” OR SU “head trauma” OR AB “head trauma” | 2,893 |
| 5 | Traumatic brain injury | TI “traumatic brain injur*” OR SU “traumatic brain injur*” OR AB “traumatic brain injur*” | 18,996 |
| 6 | TBI | TI TBI OR SU TBI OR AB TBI | 10,199 |
| 7 | Brain trauma | TI “brain trauma” OR SU “brain trauma” OR AB “brain trauma” | 471 |
| 8 | Acquired brain injury | TI “acquired brain injur*” OR SU “acquired brain injur*” OR AB “acquired brain injur*” | 2,350 |
| 9 | ABI | TI ABI OR SU ABI OR AB ABI | 2,561 |
| 10 | Brain damage | TI “brain damage*” OR SU “brain damage*” OR AB “brain damage*” | 4,792 |
| 11 | Brain infarction | TI “brain infarction*” OR SU “brain infarction*” OR AB “brain infarction*” | 593 |
| 12 | Cerebral infarction | TI “cerebral infarction” OR SU “cerebral infarction” OR AB “cerebral infarction” | 3,350 |
| 13 | Apoplexia | TI apoplex* OR SU apoplex* OR AB apoplex* | 379 |
| 14 | Neurological impairment | TI “neurological impairment*” OR SU “neurological impairment*” OR AB “neurological impairment*” | 1,508 |
| 15 | Central nervous system neoplasm | TI “central nervous system neoplasm*” OR SU “central nervous system neoplasm*” OR AB “central nervous system neoplasm*” | 2,039 |
| 16 | Central nervous system tumor | TI “central nervous system tumor*” OR SU “central nervous system tumor*” OR AB “central nervous system tumor*” | 507 |
| 17 | Cerebral hemorrhage | TI “cerebral hemorrhage*” OR SU “cerebral hemorrhage*” OR AB “cerebral hemorrhage*” | 8,375 |
| 18 | Cerebrovascular accident | TI “cerebrovascular accident*” OR SU “cerebrovascular accident*” OR AB “cerebrovascular accident*” | 1,970 |
| 19 | Encephalopathy | TI encephalopath* OR SU encephalopath* OR AB encephalopath* | 11,338 |
| 20 | Subarachnoid hemorrhage | TI “subarachnoid hemorrhage*” OR SU “subarachnoid hemorrhage*” OR AB “subarachnoid hemorrhage*” | 6,923 |
| 21 |  | MM "Stroke" | 59,369 |
| 22 |  | MM "Brain Injuries" | 21,932 |
| 23 |  | MM "Head Injuries" | 5,609 |
| 24 |  | MM "Cerebral Infarction" | 148 |
| 25 |  | MM "Central Nervous System Neoplasms" | 1,639 |
| 26 |  | MM "Cerebral Hemorrhage” | 5,262 |
| 27 |  | MM "Subarachnoid Hemorrhage" | 3,943 |
| 28 |  | S1 OR S2 OR S3 OR S4 OR S5 OR S6 OR S7 OR S8 OR S9 OR S10 OR S11 OR S12 OR S13 OR S14 OR S15 OR S16 OR S17 OR S18 OR S19 OR S20 OR S21 OR S22 OR S23 OR S24 OR S25 OR S26 OR S27 | 215,110 |

**Block 2: Hope**

| **Nr.** | **Search term** | **CINAHL** | **Number of hits** |
| --- | --- | --- | --- |
| 29 | Hope | TI hope OR SU hope OR AB hope | 29,753 |
| 30 | Hopeful | TI hopeful OR SU hopeful OR AB hopeful | 1,276 |
| 31 | Hopefulness | TI hopefulness OR SU hopefulness OR AB hopefulness | 353 |
| 32 | Hopelessness | TI hopelessness OR SU hopelessness OR AB hopelessness | 3,560 |
| 33 | Despair | TI despair OR SU despair OR AB despair | 1,367 |
| 34 | Meaning making | ( TI "meaning making" OR SU "meaning making" OR AB "meaning making" ) OR ( TI "meaning-making" OR SU "meaning-making" OR AB "meaning-making" ) | 1,125 |
| 35 | Meaning making process | ( TI "meaning making process*" OR SU "meaning making process*" OR AB "meaning making process*" ) OR ( TI "meaning-making process*" OR SU "meaning-making process*" OR AB "meaning-making process*" ) | 147 |
| 36 | Optimism | TI optimism OR SU optimism OR AB optimism | 6,973 |
| 37 | Pessimism | TI pessimism OR SU pessimism OR AB pessimism | 1,362 |
| 38 | Wish | TI wish OR SU wish OR AB wish | 13,626 |
| 39 | Wishful thinking | TI "wishful thinking" OR SU "wishful thinking" OR AB "wishful thinking" | 252 |
| 40 |  | MM "Hope" | 2,810 |
| 41 |  | MM "Hopelessness" | 499 |
| 42 |  | MM "Optimism" | 1,248 |
| 43 |  | MM "Pessimism" | 341 |
| 44 |  | S29 OR S30 OR S31 OR S32 OR S33 OR S34 OR S35 OR S36 OR S37 OR S38 OR S39 OR S40 OR S41 OR S42 OR S43 | 56,265 |

**Block 1 (Stroke) AND Block 2 (Hope):**

| 45 |  | S28 AND S44 | 1,230 |
| --- | --- | --- | --- |

**Block 3: Rehabilitation**

| **Nr.** | **Search term** | **CINAHL** | **Number of hits** |
| --- | --- | --- | --- |
| 46 | Rehabilitation | TI rehabilitation OR SU rehabilitation OR AB rehabilitation | 200,311 |
| 47 | Rehabilitation process | TI “rehabilitation process*” OR SU “rehabilitation process*” OR AB “rehabilitation process*” | 2,151 |
| 48 | Rehabilitation journey | TI "rehabilitation journey” OR SU “rehabilitation journey” OR AB “rehabilitation journey” | 30 |
| 49 | Recovery | TI recovery OR SU recovery OR AB recovery | 121,771 |
| 50 | Recovery process | TI "recovery process*” OR SU "recovery process*” OR AB "recovery process*” | 2,159 |
| 51 | Recovery journey | TI "recovery journey” OR SU "recovery journey” OR AB "recovery journey” | 144 |
| 52 |  | MM "Rehabilitation” | 12,128 |
| 53 |  | MM "Recovery" | 14,819 |
| 54 |  | S46 OR S47 OR S48 OR S49 OR S50 OR S51 OR S52 OR S53 | 301,192 |

**Block 1 (Stroke) AND Block 2 (Hope) AND Block 3 (Rehabilitation):**

| 55 |  | S28 AND S44 AND S54 | 446 |
| --- | --- | --- | --- |

| **56** |  | **S22 AND S44 AND S54**  **Limiters - Date of Publication: 20210101-20231231**  **Narrow by Language: - english.** | **31** |
| --- | --- | --- | --- |

**Search in Social Work Abstracts**

Search made the 24/01 2023

**Block 1: Stroke**

| **Nr.** | **Search term** | **Social Work Abstracts** | **Number of hits** |
| --- | --- | --- | --- |
| 1 | Stroke | TI stroke OR SU stroke OR AB stroke | 74 |
| 2 | Brain injury | TI “brain injur*” OR SU “brain injur*” OR AB “brain injur*” | 66 |
| 3 | Head injury | TI “head injur*” OR SU “head injur*” OR AB “head injur*” | 44 |
| 4 | Head trauma | TI “head trauma” OR SU “head trauma” OR AB “head trauma” | 7 |
| 5 | Traumatic brain injury | TI “traumatic brain injur*” OR SU “traumatic brain injur*” OR AB “traumatic brain injur*” | 42 |
| 6 | TBI | TI TBI OR SU TBI OR AB TBI | 15 |
| 7 | Brain trauma | TI “brain trauma” OR SU “brain trauma” OR AB “brain trauma” | 0 |
| 8 | Acquired brain injury | TI “acquired brain injur*” OR SU “acquired brain injur*” OR AB “acquired brain injur*” | 9 |
| 9 | ABI | TI ABI OR SU ABI OR AB ABI | 8 |
| 10 | Brain damage | TI “brain damage*” OR SU “brain damage*” OR AB “brain damage*” | 34 |
| 11 | Brain infarction | TI “brain infarction*” OR SU “brain infarction*” OR AB “brain infarction*” | 0 |
| 12 | Cerebral infarction | TI “cerebral infarction” OR SU “cerebral infarction” OR AB “cerebral infarction” | 0 |
| 13 | Apoplexia | TI apoplex* OR SU apoplex* OR AB apoplex* | 0 |
| 14 | Neurological impairment | TI “neurological impairment*” OR SU “neurological impairment*” OR AB “neurological impairment*” | 6 |
| 15 | Central nervous system neoplasm | TI “central nervous system neoplasm*” OR SU “central nervous system neoplasm*” OR AB “central nervous system neoplasm*” | 0 |
| 16 | Central nervous system tumor | TI “central nervous system tumor*” OR SU “central nervous system tumor*” OR AB “central nervous system tumor*” | 0 |
| 17 | Cerebral hemorrhage | TI “cerebral hemorrhage*” OR SU “cerebral hemorrhage*” OR AB “cerebral hemorrhage*” | 0 |
| 18 | Cerebrovascular accident | TI “cerebrovascular accident*” OR SU “cerebrovascular accident*” OR AB “cerebrovascular accident*” | 1 |
| 19 | Encephalopathy | TI encephalopath* OR SU encephalopath* OR AB encephalopath* | 0 |
| 20 | Subarachnoid hemorrhage | TI “subarachnoid hemorrhage*” OR SU “subarachnoid hemorrhage*” OR AB “subarachnoid hemorrhage*” | 1 |
| 21 |  | S1 OR S2 OR S3 OR S4 OR S5 OR S6 OR S7 OR S8 OR S9 OR S10 OR S11 OR S12 OR S13 OR S14 OR S15 OR S16 OR S17 OR S18 OR S19 OR S20 | 196 |

**Block 2: Hope**

| **Nr.** | **Search term** | **Social Work Abstracts** | **Number of hits** |
| --- | --- | --- | --- |
| 22 | Hope | TI hope OR SU hope OR AB hope | 640 |
| 23 | Hopeful | TI hopeful OR SU hopeful OR AB hopeful | 44 |
| 24 | Hopefulness | TI hopefulness OR SU hopefulness OR AB hopefulness | 19 |
| 25 | Hopelessness | TI hopelessness OR SU hopelessness OR AB hopelessness | 126 |
| 26 | Despair | TI despair OR SU despair OR AB despair | 84 |
| 27 | Meaning making | ( TI "meaning making" OR SU "meaning making" OR AB "meaning making" ) OR ( TI "meaning-making" OR SU "meaning-making" OR AB "meaning-making" ) | 50 |
| 28 | Meaning making process | ( TI "meaning making process*" OR SU "meaning making process*" OR AB "meaning making process*" ) OR ( TI "meaning-making process*" OR SU "meaning-making process*" OR AB "meaning-making process*" ) | 3 |
| 29 | Optimism | TI optimism OR SU optimism OR AB optimism | 156 |
| 30 | Pessimism | TI pessimism OR SU pessimism OR AB pessimism | 35 |
| 31 | Wish | TI wish OR SU wish OR AB wish | 350 |
| 32 | Wishful thinking | TI "wishful thinking" OR SU "wishful thinking" OR AB "wishful thinking" | 11 |
| 33 |  | S22 OR S23 OR S24 OR S25 OR S26 OR S27 OR S28 OR S29 OR S30 OR S31 OR S32 | 1,411 |

**Block 1 (Stroke) AND Block 2 (Hope):**

| 34 |  | S21 AND S33 | 3 |
| --- | --- | --- | --- |

**Block 3: Rehabilitation**

| **Nr.** | **Search term** | **Social Work Abstracts** | **Number of hits** |
| --- | --- | --- | --- |
| 35 | Rehabilitation | TI rehabilitation OR SU rehabilitation OR AB rehabilitation | 924 |
| 36 | Rehabilitation process | TI “rehabilitation process*” OR SU “rehabilitation process*” OR AB “rehabilitation process*” | 24 |
| 37 | Rehabilitation journey | TI "rehabilitation journey” OR SU “rehabilitation journey” OR AB “rehabilitation journey” | 0 |
| 38 | Recovery | TI recovery OR SU recovery OR AB recovery | 887 |
| 39 | Recovery process | TI "recovery process*” OR SU "recovery process*” OR AB "recovery process*” | 69 |
| 40 | Recovery journey | TI "recovery journey” OR SU "recovery journey” OR AB "recovery journey” | 3 |
| 41 |  | S35 OR S36 OR S37 OR S38 OR S39 OR S40 | 1,757 |

**Block 1 (Stroke) AND Block 2 (Hope) AND Block 3 (Rehabilitation):**

| **43** |  | **S21 AND S33 AND S41** | **0** |
| --- | --- | --- | --- |

**Search in ERIC**

Search made the 24/01 2023

**Block 1: Stroke**

| **Nr.** | **Search term** | **ERIC** | **Number of hits** |
| --- | --- | --- | --- |
| 1 | Stroke | TI stroke OR SU stroke OR AB stroke | 1,078 |
| 2 | Brain injury | TI “brain injur*” OR SU “brain injur*” OR AB “brain injur*” | 1,074 |
| 3 | Head injury | TI “head injur*” OR SU “head injur*” OR AB “head injur*” | 1,008 |
| 4 | Head trauma | TI “head trauma” OR SU “head trauma” OR AB “head trauma” | 48 |
| 5 | Traumatic brain injury | TI “traumatic brain injur*” OR SU “traumatic brain injur*” OR AB “traumatic brain injur*” | 601 |
| 6 | TBI | TI TBI OR SU TBI OR AB TBI | 350 |
| 7 | Brain trauma | TI “brain trauma” OR SU “brain trauma” OR AB “brain trauma” | 12 |
| 8 | Acquired brain injury | TI “acquired brain injur*” OR SU “acquired brain injur*” OR AB “acquired brain injur*” | 109 |
| 9 | ABI | TI ABI OR SU ABI OR AB ABI | 141 |
| 10 | Brain damage | TI “brain damage*” OR SU “brain damage*” OR AB “brain damage*” | 568 |
| 11 | Brain infarction | TI “brain infarction*” OR SU “brain infarction*” OR AB “brain infarction*” | 1 |
| 12 | Cerebral infarction | TI “cerebral infarction” OR SU “cerebral infarction” OR AB “cerebral infarction” | 5 |
| 13 | Apoplexia | TI apoplex* OR SU apoplex* OR AB apoplex* | 0 |
| 14 | Neurological impairment | TI “neurological impairment*” OR SU “neurological impairment*” OR AB “neurological impairment*” | 5,216 |
| 15 | Central nervous system neoplasm | TI “central nervous system neoplasm*” OR SU “central nervous system neoplasm*” OR AB “central nervous system neoplasm*” | 0 |
| 16 | Central nervous system tumor | TI “central nervous system tumor*” OR SU “central nervous system tumor*” OR AB “central nervous system tumor*” | 1 |
| 17 | Cerebral hemorrhage | TI “cerebral hemorrhage*” OR SU “cerebral hemorrhage*” OR AB “cerebral hemorrhage*” | 0 |
| 18 | Cerebrovascular accident | TI “cerebrovascular accident*” OR SU “cerebrovascular accident*” OR AB “cerebrovascular accident*” | 15 |
| 19 | Encephalopathy | TI encephalopath* OR SU encephalopath* OR AB encephalopath* | 47 |
| 20 | Subarachnoid hemorrhage | TI “subarachnoid hemorrhage*” OR SU “subarachnoid hemorrhage*” OR AB “subarachnoid hemorrhage*” | 4 |
| 21 |  | DE "Head Injuries" | 888 |
| 22 |  | DE "Neurological Impairments" | 5,191 |
| 23 |  | S1 OR S2 OR S3 OR S4 OR S5 OR S6 OR S7 OR S8 OR S9 OR S10 OR S11 OR S12 OR S13 OR S14 OR S15 OR S16 OR S17 OR S18 OR S19 OR S20 OR S21 OR S22 | 7,113 |

**Block 2: Hope**

| **Nr.** | **Search term** | **ERIC** | **Number of hits** |
| --- | --- | --- | --- |
| 24 | Hope | TI hope OR SU hope OR AB hope | 12,772 |
| 25 | Hopeful | TI hopeful OR SU hopeful OR AB hopeful | 711 |
| 26 | Hopefulness | TI hopefulness OR SU hopefulness OR AB hopefulness | 80 |
| 27 | Hopelessness | TI hopelessness OR SU hopelessness OR AB hopelessness | 668 |
| 28 | Despair | TI despair OR SU despair OR AB despair | 545 |
| 29 | Meaning making | ( TI "meaning making" OR SU "meaning making" OR AB "meaning making" ) OR ( TI "meaning-making" OR SU "meaning-making" OR AB "meaning-making" ) | 2,433 |
| 30 | Meaning making process | ( TI "meaning making process*" OR SU "meaning making process*" OR AB "meaning making process*" ) OR ( TI "meaning-making process*" OR SU "meaning-making process*" OR AB "meaning-making process*" ) | 253 |
| 31 | Optimism | TI optimism OR SU optimism OR AB optimism | 1,904 |
| 32 | Pessimism | TI pessimism OR SU pessimism OR AB pessimism | 349 |
| 33 | Wish | TI wish OR SU wish OR AB wish | 6,811 |
| 34 | Wishful thinking | TI "wishful thinking" OR SU "wishful thinking" OR AB "wishful thinking" | 96 |
| 35 |  | S24 OR S25 OR S26 OR S27 OR S28 OR S29 OR S30 OR S31 OR S32 OR S33 OR S34 | 25,394 |

**Block 1 (Stroke) AND Block 2 (Hope):**

| 36 |  | S23 AND S35 | 70 |
| --- | --- | --- | --- |

**Block 3: Rehabilitation**

| **Nr.** | **Search term** | **ERIC** | **Number of hits** |
| --- | --- | --- | --- |
| 37 | Rehabilitation | TI rehabilitation OR SU rehabilitation OR AB rehabilitation | 15,479 |
| 38 | Rehabilitation process | TI “rehabilitation process*” OR SU “rehabilitation process*” OR AB “rehabilitation process*” | 207 |
| 39 | Rehabilitation journey | TI "rehabilitation journey” OR SU “rehabilitation journey” OR AB “rehabilitation journey” | 0 |
| 40 | Recovery | TI recovery OR SU recovery OR AB recovery | 5,732 |
| 41 | Recovery process | TI "recovery process*” OR SU "recovery process*” OR AB "recovery process*” | 127 |
| 42 | Recovery journey | TI "recovery journey” OR SU "recovery journey” OR AB "recovery journey” | 5 |
| 43 |  | DE "Rehabilitation" | 3,050 |
| 44 |  | S37 OR S38 OR S39 OR S40 OR S41 OR S42 OR S43 | 20,775 |

**Block 1 (Stroke) AND Block 2 (Hope) AND Block 3 (Rehabilitation):**

| 45 |  | S23 AND S35 AND S44 | 19 |
| --- | --- | --- | --- |

| **46** |  | **S23 AND S35 AND S44**  **Limiters - Scholarly (Peer Reviewed) Journals; Date of Publication: 20211201-20231231**  **Narrow by Language: - English** | **1** |
| --- | --- | --- | --- |

**Search in Web of Science**

Search made the 24/01 2023

**Block 1: Stroke**

| **Nr.** | **Search term** | **Web of Science** | **Number of hits** |
| --- | --- | --- | --- |
| 1 | Stroke | TS=(stroke) | 406,212 |
| 2 | Brain injury | TS=(“brain injur*”) | 117,830 |
| 3 | Head injury | TS=(“head injur*”) | 39,708 |
| 4 | Head trauma | TS=(“head trauma”) | 10,920 |
| 5 | Traumatic brain injury | TS=(“traumatic brain injur*”) | 68,148 |
| 6 | TBI | TS=(TBI) | 32,542 |
| 7 | Brain trauma | TS=(“brain trauma”) | 3,035 |
| 8 | Acquired brain injury | TS=(“acquired brain injur*”) | 3,830 |
| 9 | ABI | TS=(ABI) | 9,290 |
| 10 | Brain damage | TS=(“brain damage*”) | 28,908 |
| 11 | Brain infarction | TS=(“brain infarction*”) | 3,678 |
| 12 | Cerebral infarction | TS=(“cerebral infarction”) | 18,850 |
| 13 | Apoplexia | TS=(apoplex*) | 2,597 |
| 14 | Neurological impairment | TS=(“neurological impairment*”) | 5,414 |
| 15 | Central nervous system neoplasm | TS=( “central nervous system neoplasm*”) | 569 |
| 16 | Central nervous system tumor | TS=( “central nervous system tumor*”) | 2,873 |
| 17 | Cerebral hemorrhage | TS=(“cerebral hemorrhage*”) | 6,570 |
| 18 | Cerebrovascular accident | TS=(“cerebrovascular accident*”) | 7,478 |
| 19 | Encephalopathy | TS=(encephalopath*) | 68,717 |
| 20 | Subarachnoid hemorrhage | TS=(“subarachnoid hemorrhage*”) | 30,953 |
| 21 |  | #1 OR #2 OR #3 OR #4 OR #5 OR #6 OR #7 OR #8 OR #9 OR #10 OR #11 OR #12 OR #13 OR #14 OR #15 OR #16 OR #17 OR #18 OR #19 OR #20 | 669,872 |

**Block 2: Hope**

| **Nr.** | **Search term** | **Web of Science** | **Number of hits** |
| --- | --- | --- | --- |
| 22 | Hope | TS=(hope) | 137,964 |
| 23 | Hopeful | TS=(hopeful) | 5,784 |
| 24 | Hopefulness | TS=(hopefulness) | 639 |
| 25 | Hopelessness | TS=(hopelessness) | 7,472 |
| 26 | Despair | TS=(despair) | 6,349 |
| 27 | Meaning making | TS=("meaning making" OR TS "meaning-making") | 5,987 |
| 28 | Meaning making process | TS=("meaning making process*" OR TS "meaning making process*") | 734 |
| 29 | Optimism | TS=(optimism) | 21,939 |
| 30 | Pessimism | TS=(pessimism) | 5,229 |
| 31 | Wish | TS=(wish) | 60,866 |
| 32 | Wishful thinking | TS=("wishful thinking") | 1,173 |
| 33 |  | #22 OR #23 OR #24 OR #25 OR #26 OR #27 OR #28 OR #29 OR #30 OR #31 OR #32 | 243,923 |

**Block 1 (Stroke) AND Block 2 (Hope):**

| 34 |  | S21 AND S33 | 3,339 |
| --- | --- | --- | --- |

**Block 3: Rehabilitation**

| **Nr.** | **Search term** | **Web of Science** | **Number of hits** |
| --- | --- | --- | --- |
| 35 | Rehabilitation | TS=(rehabilitation) | 239,694 |
| 36 | Rehabilation process | TS=(“rehabilitation process*”) | 3,109 |
| 37 | Rehabilitation journey | TS=("rehabilitation journey”) | 31 |
| 38 | Recovery | TS=(recovery) | 924,350 |
| 39 | Recovery process | TS=("recovery process*”) | 13,950 |
| 40 | Recovery journey | TS=("recovery journey”) | 175 |
| 41 |  | #35 OR #36 OR #37 OR #38 OR #39 OR #40 | 1,133,231 |

**Block 1 (Stroke) AND Block 2 (Hope) AND Block 3 (Rehabilitation):**

| 42 |  | #21 AND #33 AND #41 | 808 |
| --- | --- | --- | --- |

| **43** |  | **#21 AND #33 AND #41**  **Limiters - Date of Publication: 20210101-20231231**  **Narrow by Language: - english.** | **149** |
| --- | --- | --- | --- |

**Search in MEDLINE**

Search made the 24/01 2023

**Block 1: Stroke**

| **Nr.** | **Search term** | **MEDLINE** | **Number of hits** |
| --- | --- | --- | --- |
| 1 | Stroke | TS=(stroke) | 336,724 |
| 2 | Brain injury | TS=(“brain injur*”) | 108,709 |
| 3 | Head injury | TS=(“head injur*”) | 29,390 |
| 4 | Head trauma | TS=(“head trauma”) | 10,443 |
| 5 | Traumatic brain injury | TS=(”traumatic brain injur*”) | 46,817 |
| 6 | TBI | TS=(TBI) | 30,380 |
| 7 | Brain trauma | TS=(“brain trauma”) | 2,935 |
| 8 | Acquired brain injury | TS=(“acquired brain injur*”) | 2,873 |
| 9 | ABI | TS=(ABI) | 8,194 |
| 10 | Brain damage | TS=(“brain damage*”) | 35,052 |
| 11 | Brain infarction | TS=(“brain infarction*”) | 7,265 |
| 12 | Cerebral infarction | TS=(“cerebral infarction”) | 34,193 |
| 13 | Apoplexia | TS=(apoplex*) | 3,705 |
| 14 | Neurological impairment | TS=(“neurological impairment*”) | 5,928 |
| 15 | Central nervous system neoplasm | TS=( “central nervous system neoplasm*”) | 8,182 |
| 16 | Central nervous system tumor | TS=( “central nervous system tumor*”) | 2,872 |
| 17 | Cerebral hemorrhage | TS=(“cerebral hemorrhage*”) | 39,750 |
| 18 | Cerebrovascular accident | TS=(“cerebrovascular accident*”) | 8,140 |
| 19 | Encephalopathy | TS=(encephalopath*) | 65,465 |
| 20 | Subarachnoid hemorrhage | TS=(“subarachnoid hemorrhage*”) | 33,289 |
| 21 |  | MH=(Stroke) | 127,498 |
| 22 |  | MH=(Brain Injuries) | 55,441 |
| 23 |  | MH=(Head Injuries, Closed) | 3,201 |
| 24 |  | MH=(Brain Injuries, Traumatic) | 11,960 |
| 25 |  | MH=(Brain Damage, Chronic) | 13,360 |
| 26 |  | MH=(Brain Infarction) | 4,830 |
| 27 |  | MH=(Cerebral Infarction) | 23,508 |
| 28 |  | MH=(Cerebral Hemorrhage) | 36,274 |
| 29 |  | MH=(Cerebral Hemorrhage, Traumatic) | 361 |
| 30 |  | MH=(Subarachnoid Hemorrhage) | 23,360 |
| 31 |  | MH=(Subarachnoid Hemorrhage, Traumatic) | 294 |
| 32 |  | #1 OR #2 OR #3 OR #4 OR #5 OR #6 OR #7 OR #8 OR #9 OR #10 OR #11 OR #12 OR #13 OR #14 OR #15 OR #16 OR #17 OR #18 OR #19 OR #20 OR #21 OR #22 OR #23 OR #24 OR #25 OR #26 OR #27 OR #28 OR #29 OR #30 OR #31 | 670,957 |

**Block 2: Hope**

| **Nr.** | **Search term** | **MEDLINE** | **Number of hits** |
| --- | --- | --- | --- |
| 33 | Hope | TS=(hope) | 90,487 |
| 34 | Hopeful | TS=(hopeful) | 3,293 |
| 35 | Hopefulness | TS=(hopefulness) | 471 |
| 36 | Hopelessness | TS=(hopelessness) | 5,007 |
| 37 | Despair | TS=(despair) | 3,078 |
| 38 | Meaning making | TS=("meaning making" OR TS "meaning-making") | 1,351 |
| 39 | Meaning making process | TS=("meaning making process*" OR TS "meaning making process*") | 189 |
| 40 | Optimism | TS=(optimism) | 11,007 |
| 41 | Pessimism | TS=(pessimism) | 2,004 |
| 42 | Wish | TS=(wish) | 44,586 |
| 43 | Wishful thinking | TS=("wishful thinking") | 612 |
| 44 |  | MH=(Hope) | 1,626 |
| 45 |  | MH=(Optimism) | 1,026 |
| 46 |  | MH=(Pessimism) | 364 |
| 47 |  | #33 OR #34 OR #35 OR #36 OR #37 OR #38 OR #39 OR #40 OR #41 OR #42 OR #43 OR #44 OR #45 OR #46 | 157,168 |

**Block 1 (Stroke) AND Block 2 (Hope):**

| 48 |  | S32 AND S47 | 3,537 |
| --- | --- | --- | --- |

**Block 3: Rehabilitation**

| **Nr.** | **Search term** | **MEDLINE** | **Number of hits** |
| --- | --- | --- | --- |
| 49 | Rehabilitation | TS=(rehabilitation) | 360,052 |
| 50 | Rehabilitation process | TS=(“rehabilitation process*”) | 3,020 |
| 51 | Rehabilitation journey | TS=("rehabilitation journey”) | 29 |
| 52 | Recovery | TS=(recovery) | 603,034 |
| 53 | Recovery process | TS=("recovery process*”) | 6,554 |
| 54 | Recovery journey | TS=("recovery journey”) | 144 |
| 55 |  | MH=(Rehabilitation) | 18,663 |
| 56 |  | MH=(Stroke Rehabilitation) | 16,959 |
| 57 |  | MH=(Recovery of Function) | 58,871 |
| 58 |  | #49 OR #50 OR #51 OR #52 OR #53 OR #54 OR #55 OR #56 OR #57 | 925,558 |

**Block 1 (Stroke) AND Block 2 (Hope) AND Block 3 (Rehabilitation):**

| 59 |  | #32 AND #47 AND #58 | 783 |
| --- | --- | --- | --- |

| **60** |  | **#32 AND #47 AND #58**  **Limiters - Date of Publication: 20210101-20231231**  **Narrow by Language: - english.** | **122** |
| --- | --- | --- | --- |
